# Glial cells are functionally impaired in juvenile neuronal ceroid lipofuscinosis and detrimental to neurons

**DOI:** 10.1186/s40478-017-0476-y

**Published:** 2017-10-17

**Authors:** Lotta Parviainen, Sybille Dihanich, Greg W. Anderson, Andrew M. Wong, Helen R. Brooks, Rosella Abeti, Payam Rezaie, Giovanna Lalli, Simon Pope, Simon J. Heales, Hannah M. Mitchison, Brenda P. Williams, Jonathan D. Cooper

**Affiliations:** 1 Department of Basic and Clinical Neuroscience, King’s College London, Institute of Psychiatry, Psychology & Neuroscience, Maurice Wohl Clinical Neuroscience Institute, 5 Cutcombe Road, London, SE5 9RX UK; 2 Department of Molecular Neuroscience, Institute of Neurology, University College London, Queen’s Square, London, WC1N, 3BG UK; 3 Department of Life, Health and Chemical Sciences, The Open University, Walton Hall, Milton Keynes, MK7 6AA UK; 4 Wolfson Centre for Age-Related Diseases, King’s College London, London, SE1 1UL UK; 5 Genetics and Genomic Medicine UCL Great Ormond Street Institute of Child Health, University College London, 30 Guilford Street, London, WC1N 1EH UK; 6 Department of Pediatrics, Harbor-UCLA Medical Center, Los Angeles Biomedical Research Institute and David Geffen School of Medicine UCLA, 1124 West Carson Street, Hanley Hardison Building, Torrance, CA 90502 USA

**Keywords:** Juvenile batten disease, CLN3 disease, Neuronal ceroid lipofuscinosis, Neuron-glial interactions, Astrocyte and microglial dysfunction

## Abstract

The neuronal ceroid lipofuscinoses (NCLs or Batten disease) are a group of inherited, fatal neurodegenerative disorders of childhood. In these disorders, glial (microglial and astrocyte) activation typically occurs early in disease progression and predicts where neuron loss subsequently occurs. We have found that in the most common juvenile form of NCL (CLN3 disease or JNCL) this glial response is less pronounced in both mouse models and human autopsy material, with the morphological transformation of both astrocytes and microglia severely attenuated or delayed. To investigate their properties, we isolated glia and neurons from *Cln3-*deficient mice and studied their basic biology in culture. Upon stimulation, both *Cln3*-deficient astrocytes and microglia also showed an attenuated ability to transform morphologically, and an altered protein secretion profile. These defects were more pronounced in astrocytes, including the reduced secretion of a range of neuroprotective factors, mitogens, chemokines and cytokines, in addition to impaired calcium signalling and glutamate clearance. *Cln3*-deficient neurons also displayed an abnormal organization of their neurites. Most importantly, using a co-culture system, *Cln3-*deficient astrocytes and microglia had a negative impact on the survival and morphology of both *Cln3-*deficient and wildtype neurons, but these effects were largely reversed by growing mutant neurons with healthy glia. These data provide evidence that CLN3 disease astrocytes are functionally compromised. Together with microglia, they may play an active role in neuron loss in this disorder and can be considered as potential targets for therapeutic interventions.

**Electronic supplementary material:**

The online version of this article (10.1186/s40478-017-0476-y) contains supplementary material, which is available to authorized users.

## Introduction

The neuronal ceroid lipofuscinoses (NCLs) or Batten disease are a group of fatal lysosomal storage disorders, and are collectively the most common cause of childhood dementia [91]. Each form of NCL is caused by mutations in a different gene, which determines the age of disease onset, symptoms and rate of disease progression, but all are fatal after a period of prolonged disability [79, 96]. Very little is known about how mutations in these genes lead to devastating effects upon the brain, but these diseases share common pathological features, including accumulation of autofluorescent storage material within the lysosome and profound neuronal loss [2, 25, 63].

Clues to understanding NCL pathogenesis have come from studying mouse models [13, 23, 24, 82]. Although neuron loss is widespread at the end stages of disease, there is remarkable selectivity in its earlier stages with these effects being most prominent within the thalamocortical system and the cerebellum, as reviewed in [24, 25, 63]. However, no direct relationship has been found between this pattern of neuron loss and storage material accumulation [24, 25, 63]. Instead, localized glial activation consistently occurs early in NCL disease progression, and its distribution accurately predicts where neuron loss subsequently occurs, as reviewed in [25, 63]. There is also evidence from human autopsy material that neuron loss is greatest where astrocytosis and microglial activation is most pronounced [2, 37, 90].

In the most common juvenile form of NCL (JNCL or CLN3 disease) the activation of both astrocytes and microglia appears to be attenuated compared to other earlier onset forms of NCL [68, 69, 90]. We have explored this issue in more detail in this study, as such observations raise the possibility that normal glial function may be compromised by *CLN3* deficiency. Since both astrocytes [66, 85] and microglia [5] are crucial for proper neuron function and survival, as well as playing a pivotal role in the pathogenesis of CNS diseases, any deficits in the biology of these cells could significantly impact upon neuronal health. Indeed, recent evidence suggests that this could be the case in CLN3 disease, with a potential influence of both microglia and astrocytes [16, 99]. There is also evidence in CNS disease and injury that astrocytes may be primed by microglia to directly harm neurons [49], raising the possibility that glia may actively contribute to the pathogenesis of a range of disorders. Furthermore, astrocyte dysfunction is suggested to trigger neurodegeneration specifically in lysosomal storage disorders, for example in multiple sulfatase deficiency [28], and in Niemann-Pick disease type C [21].

In this study, we have explored the role of glia in CLN3 disease using primary cultures of microglia, astrocytes and neurons derived from *Cln3*-deficient mice. Grown in isolation, both types of glia responded atypically to stimulation and displayed altered protein secretion profiles. These differences were more profound in astrocytes, which displayed a disrupted actin and intermediate filament cytoskeleton and an impaired ability to propagate a calcium signal and clear glutamate, suggesting that neuron-glial communication may be impaired in the JNCL brain. Cortical neurons from these mice displayed altered neurite branching, suggesting neurons are also compromised by Cln3 deficiency. In a mixed glial-neuron co-culture system, we found that *Cln3*-deficient glial cells had a significant negative impact upon the survival and morphology of both *Cln3*-deficient and wild type neurons, but that the defects found in mutant neurons could be markedly improved by the presence of healthy astrocytes and microglia.

These findings provide further new information on how both glia and neurons are compromised in this disorder and the negative role that glial cells appear to play in the pathogenesis of CLN3 disease, and also highlight astrocytes and microglia as novel potential targets for future therapeutic approaches.

## Materials and methods

### Animals

Homozygous *Cln3* ^*Δex1–6*^ mice (*Cln3* ^*−/−*^) were used as a model of CLN3 disease [56] and cells isolated from early postnatal mice for tissue culture, as described below, and were also assessed histologically. For histological comparisons of the level of glial activation, homozygous *Tpp-1*-deficient mice (*Tpp-1* ^*−/−*^) were used as a model of CLN2 disease (Late Infantile NCL) [84]. Wild type (WT) mice on the same strain (C57BL/6 J) background were used as controls. All animal housekeeping and procedures were carried out according to the UK Scientific Procedures (Animals) Act (1986). *Cln3* ^*−/−*^ mice were analyzed histologically at 6.5 months (early symptomatic), 12 months (disease mid stage), and 22 months of age (severely affected), and *Tpp-1* ^*−/−*^ mice histologically at 4 months of age (severely affected).

### Human tissues

Human specimens were obtained from the Human Brain and Spinal Fluid Resource Centre, Los Angeles and the MRC London Neurodegenerative Diseases Brain Bank, Institute of Psychiatry, King’s College London following routine autopsies of NCL patients with informed written consent from their families. At autopsy, tissues were fixed immediately by immersion in 4% neutral buffered formaldehyde and subsequently processed and embedded in paraffin wax. These cases included NCL patients with CLN2 (*n* = 2; 6 years old Female, 26 years Male), CLN3 (n = 2; 20 years old Male, 24 years old Female), neurologically normal controls (n = 2 ages 25 years Male, 26 years Female). Study protocols for the use of human material were approved by the Ethical Research Committees of the Institute of Psychiatry (approval numbers 223/00, 181/02).

### Histological analysis

To investigate glial activation in the mouse brain, frozen sections from *Cln3* ^*−/−*^, *Tpp-1* ^*−/−*^ and WT mice were prepared as previously described [8, 40, 68, 69]. To investigate glial activation in the human NCL brain, paraffin-embedded tissue blocks were prepared from the primary visual cortical region of human CLN2 and CLN3 autopsy tissue (*n* = 2 for each type of NCL), and cut into 8 μm sections, as previously described [18, 90]. Both mouse and human sections containing the primary visual cortex were immunostained with antibodies to glial fibrillary acidic protein (GFAP, 1:1000 for mouse tissue, 1:5000 for human tissue, rabbit polyclonal, Dako) to identify activated astrocytes and Cluster of Differentiation 68 (CD68, 1:150, Rat monoclonal, Serotec) to identify activated microglia [54, 59, 71]. Immunostaining was detected using *VECTASTAIN Elite ABC* Reagent (Vector Laboratories) and DAB substrate (Sigma) and human sections counterstained with hematoxylin [18, 90].

### Tissue culture

#### Glial cultures

Mixed glial cells were isolated from *post-natal* day 1–4 (P1-P4) *Cln3* ^*−/−*^ or WT mouse cerebral cortices, as previously described [52, 97]. Once these cultures reached confluence they were composed of a base layer of non-dividing astrocytes and an upper layer of dividing microglia and a few oligodendrocytes. Microglial cultures were isolated from these P2-P4 mixed glial cultures by shaking at 180 rpm for 10-12 h at 37°C in a humidified incubator 5% CO_2_ [97]. Cells were harvested, re-suspended in RPMI 1640 (Gibco, Invitrogen) supplemented with penicillin/streptomycin (100 U/mL, 100 mg/mL, Sigma, UK), 5% FBS (Gibco, Invitrogen) and 2 mM L-Glutamine (Sigma), plus macrophage colony-stimulating factor (M-CSF, 10 ng/ml) and granulocyte macrophage colony-stimulating factor (GM-CSF, 10 ng/ml) (both R&D Systems, Minneapolis, MN) to promote proliferation [35, 87], then plated at a concentration of 1–2 × 10^5^ cells per flask on poly-D-lysine (PDL, 25μg/ml, Sigma) coated T25 (Corning, Costar) flasks. To generate astrocyte cultures (from P1-P2 mice), microglia were removed, as described above, and these confluent astrocyte monolayers were treated with Ara-C (Arabinofuranosyl Cytidine, 2 × 10^−5^ mol/l) for 7 days to abolish any remaining dividing cells. As such, at the start of all experiments described, these glial cells had been cultured for approximately 21 days (astrocytes) or 12–14 days (microglia). All cultures used in these studies exhibited a purity of >98% (astrocytes) or >99% (microglia) at one week after plating, as determined by immunofluorescence staining, but their composition may subsequently vary over time under some culture conditions.

#### Neuronal cultures

Cells were isolated from P0 WT or *Cln3* ^*−/−*^ mouse cerebral cortices as described previously [10, 11] and plated on PDL coated (50 μg/ml), 13 mm glass coverslips (VWR) in 24 well plates (Corning, Costar) at a concentration of 2.5–3 × 10^5^ cells per coverslip.

#### Neuron-glia co-cultures

Co-cultures were generated by plating 50,000 mixed WT or *Cln3* ^*−/−*^ glial cells from 3 to 4 week old cultures directly on top of 7 day old neuronal cultures.

### Pharmacological activation of glial cells

Microglial cells were activated by exposure to lipopolysaccharide (LPS, 1 μg/ml LPS, Sigma), while astrocytes were activated by exposure to LPS plus interferon-gamma (IFN-γ, 100 U/ml, Thermo Scientific) [12, 14]. The ability of mutant and WT glia to respond similarly to LPS and IFN-γ was assessed by studying the nuclear translocation of the downstream phosphorylated proteins, NF-κβ subunit P65 (P-P65) [22] or STAT1 (P-STAT1) [39] respectively, using phospho-specific primary antibodies (P-P65, 1:100; P-STAT1, 1:50, both from Cell Signaling).

### Immunofluorescence staining

Cultures were immunostained using standard protocols (see [9]). Where appropriate, nuclei were counterstained with DAPI (4′-6-Diamidino-2-phenylindole, 0.5-1 μg/ml, Sigma) and coverslips mounted using either Fluoromount G or Prolong gold (Southern Biotech). The composition of all cultures was assessed using cell-type specific markers. GFAP (rabbit polyclonal, 1:500, Dako) or glutamate synthetase (rabbit polyclonal, 1:500, Abcam) was used to identify astrocytes, O4 (monoclonal antibody, 1:100, Covance) to identify oligodendrocytes, CD68 (Rat monoclonal, 1:500, Serotec) to identify microglia and MAP2 (monoclonal antibody, 1:1000, Abcam) and/or NeuN (monoclonal antibody, 1:100, Chemicon) to identify neurons. For cytoskeletal analysis, phalloidin was used to visualize F-actin filaments and α- and β- tubulin antibodies to visualize microtubular organization (monoclonal and polyclonal antibodies respectively, both from Sigma and used at 1:1000). All secondary antibodies were obtained from Invitrogen, and used at a dilution of 1:1000 (Alexa 488, 546, 633 and biotinylated antibodies) or 1:5000 (Alexa 790, 680). Immunofluorescently stained cells were visualized using a Zeiss AxioImager Z1 fluorescence microscope (Carl Zeiss, Ltd) with a monochrome AxioCamMR3 camera using *AxioVision* 4.8. Imaging software (Carl Zeiss, Welwyn Garden City).

### Cell death assays

The overall cytotoxicity in co-cultures was evaluated by measuring lactate dehydrogenase (LDH) release using a Cytotox 96 assay kit (Promega) according to manufacturer’s instructions. Total LDH content (100% LDH) was determined by lysing cultures in 0.1% Triton X-100 for 30 min, and LDH release from cells was expressed as a percentage of total LDH (%LDH) in each sample. To reveal the identity of the cells undergoing cell death, a live/dead fixable cellular marker conjugated to a red fluorochrome (Invitrogen) was used, according to the manufacturer’s instructions, in association with relevant cell-type specific markers.

### Assessment of morphological changes following activation

Astrocyte cultures were immunostained with GFAP and images of 10 random fields of cells, whose processes were not overlapping, were taken and cell soma size measured using *ImageJ* (National Institutes of Health, Bethesda, MD). The average cell soma size of *Cln3* ^*−/−*^ astrocytes was normalized to the corresponding values from WT astrocytes. To assess the morphological response of microglia to activation, cells were classified into 3 subcategories [98]: type 1 cells – microglia with extended processes (non-activated); type 2 cells – microglia with retracted processes (partly activated); type 3 cells– rounded cells with a small soma (fully activated), and the percentage of each morphological type present (determined from counting 10 random fields per culture) was calculated for each culture condition.

### Protein secretion analysis

The quantitative analysis of the levels of proteins secreted by *Cln3* ^*−/−*^ and WT glial cells grown under basal conditions and at various time points (between 6 h and 96 h) after activation with LPS/IFNγ was carried out by Myriad RBM (Austin, TX, USA, *RodentMAP version 2.0* cytokine analysis). Simultaneous analysis of 59 different proteins was carried out on three different biological samples for each treatment per genotype using an automated quantification system. The values obtained were normalized to the relative number of cells in the culture from which the medium was collected, as determined by counting DAPI stained nuclei.

### Glutathione measurements

The intracellular levels of reduced (GSH) and oxidized (GSSG) glutathione was determined in WT and *Cln3* ^*−/−*^astrocytes. Samples for these measurements were generated from stimulated (for 24 or 48 h) and non-stimulated cultures by trypsinization and resuspension of cells in 300μl of isolation medium (320 mM sucrose, 10 mM Tris, 1 mM EDTA, pH 7.4), and the sample split into two for testing. One half was used to quantify GSH levels by separating this antioxidant from other components within the sample using reverse-phase high performance liquid chromatography (HPLC) followed by detection using an electrochemical method [33]. The GSH levels obtained were normalized to the total amount of protein, as determined using a Lowry protein assay (Thermo Scientific). The other half of the sample was used to determine the presence of GSSG. This was carried out by treating samples with glutathione reductase (GR) in the presence of reduced nicotinamide adenosine dinucleotide phosphatase (NADPH) to convert GSSG to GSH [86], and the level determined by HPLC as before. This gives a measure of the total glutathione within the cell. The difference between total glutathione concentration and GSH concentration was then used to calculate the concentration of GSSG. Finally, glutathione levels in the culture medium were measured using the GSH-Glo glutathione assay kit (Promega), according to manufacturer’s instructions. In some experiments, the effect of actin depolymerisation on glutathione secretion was assessed by treating WT astrocytes with Cytochalasin D (1 μM), an inhibitor of actin polymerization.

### Intracellular calcium measurements

Fluctuations in the levels of intracellular Ca^2+^ ([Ca^2+^]_i_) were examined to determine the ability of WT and *Cln3* ^*−/−*^ astrocytes to generate calcium waves when exposed to ATP (100 μM) as described previously [60]. To measure intracellular Ca^2+^ levels, cells were loaded with 5 μM of Fura-2-acetoxymethyl ester (Fura-2 AM, Invitrogen), which is a membrane permeable derivative of the ratiometric calcium indicator Fura-2, for 30 min at room temperature and excess reagent removed by washing. Fluorescence measurements were carried out at room temperature using an epifluorescence inverted microscope equipped with a 20X fluorite objective over a 30–45 min period. [Ca^2+^]_i_ was monitored in single cells, with the excitation light provided by a Xenon arc lamp, using a monochromator (Cairn Research) to excite fluorescence sequentially at 340, 380 nm (all at 10 nm bandwidth). Using a long pass filter from 510 nm, the emitted fluorescence light was reflected and then transferred to a frame transfer cooled CCD camera (Hamamatsu Orca ER).

### Glutamate uptake assay

The glutamate clearance capacity of WT and *Cln3* ^*−/−*^ astrocytes was determined using a Glutamate Assay kit (Abcam), according to manufacturer’s instructions. Values were normalized to the amount of total protein in each sample, determined using a BCA protein assay kit (Thermo Scientific).

### Cell mobility assay

The ability of *Cln3* ^*−/−*^ astrocytes to migrate was assessed by performing a scratch wound assay. A scratch was made in confluent astrocyte cultures grown on Essen Image Lock 24-well plates using an Essen Wound-maker, generating an 800–900 μm wide cell-free region. Cultures were then placed in the *IncuCyte* live cell imaging system (Essen) and the wound width measured every hour for 24 h [62]. The rate of migration was obtained by measuring the width of the existing wound over time.

### Neurite complexity measurements

Neurite complexity was analyzed in P0 cortical neuron cultures from WT and *Cln3* ^*−/−*^ mice after 7 DIV using *ImageJ* software to analyze immunofluorescence images of MAP2-positive cortical neurons, measuring 40 cells per genotype per experiment. The number of primary, secondary, and tertiary neurites present on each neuron was counted, the area of its cell soma measured, together and the total length of all primary neurites and the length of the longest primary neurite (assumed to represent the axon). Similar measurements of neurite complexity and soma size were also obtained from neurons co-cultured with glial cells.

### Statistics

All quantitative data was collected using Microsoft *Excel* spreadsheets, and analyzed using *Graphpad PRISM*. Where appropriate the data were normalized to values from untreated WT cultures. Most frequently, to allow comparisons of groups, one-way ANOVA with Bonferroni correction was used to test for statistical significance. However, when two groups were compared with each other a Student’s T-test was used. In general, three technical replicates were used, and independent experiments were repeated at least three times (unless otherwise stated). Data was presented as mean ± SEM and changes were considered significant with a *p*-value of ≤0.05. *P*-values ≤0.05 marked with *, *P*-values ≤0.01 marked with **, *P*-value ≤0.001 marked with ***.

## Results

### Attenuated glial response in human CLN3 disease

In moderately affected *Cln3* ^*−/−*^ mice the reactive response of glia, judged by hallmark morphological changes, appears attenuated compared to earlier onset forms of NCL [68, 69]. To investigate this possibility further we extended our analysis to more aged and severely affected *Cln3* ^*−/−*^ mice, and also investigated the extent of glial activation in the same cortical region in human CLN3 disease autopsy tissue.

**Fig. 1:**
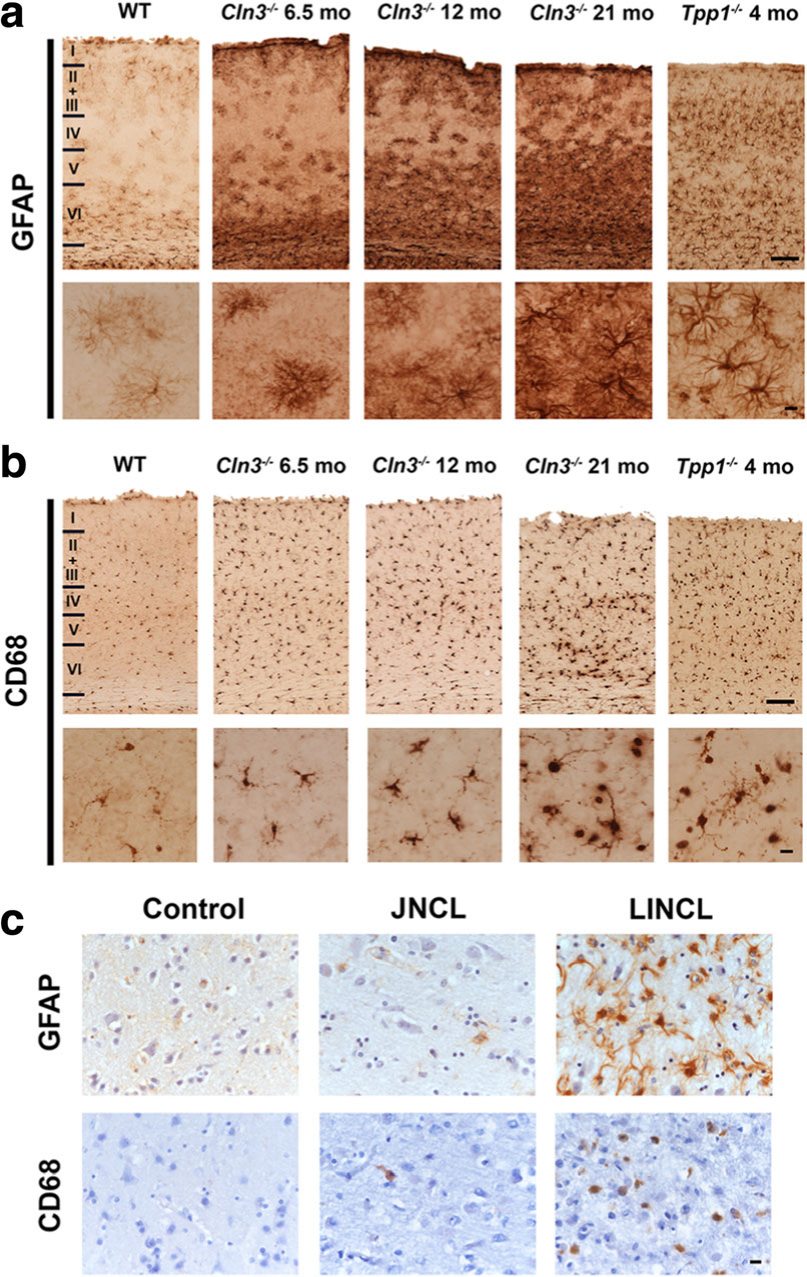
Attenuated glial responses in *Cln3* ^*−/−*^ mouse tissue and in human JNCL. Cortical sections from wild type (WT), *Cln3* ^*−/−*^ and *Tpp-1* ^*−/−*^ mice (**a**, **b**) or from LINCL and JNCL human cases (**c**) were immunostained with Glial Fibrillary Acid Protein (GFAP) or Cluster of Differentiation 68 (CD68) to investigate the level of reactive astrocytosis or microglial activation, respectively. Compared to the very low level of glial activation present in WT mice (shown at 6.5 months of age, but changes very little over time), marked astrocytosis (**a**) and microglial activation (**b**) was apparent in severely affected 4-month-old *Tpp-1* ^*−/−*^ mouse sections, with hypertrophied astrocytes with intense GFAP immunostaining and thickened processes, and morphologically transformed microglia with intense CD68 immunostaining being observed. **a** In contrast, astrocytosis appeared different in nature in the *Cln3* ^*−/−*^ cortex, and although many astrocytes displayed intense GFAP immunoreactivity, especially in the deeper laminae, most these astrocytes still retained many thin processes reminiscent of protoplasmic astrocytes. Even at the end stages of the disease (21-month-old *Cln3* ^*−/−*^ mice) very few astrocytes became fully hypertrophied. **b** A similar attenuated morphological transformation of microglial cells was also apparent in the cortex of *Cln3* ^*−/−*^ mice. Although microglia became more intensely CD68 immunoreactive and more swollen with increased age, many of these cells retaining long branched processes, even in 21-month-old *Cln3* ^*−/−*^ mice. (**c**) Reactive astrocytosis was observed in both human INCL and JNCL (**c**), but to very different extents. In LINCL cases astrocytes were intensely immunostained, with hypertrophied cell bodies and numerous thickened processes. In JNCL cases GFAP immunostaining was paler and far fewer, less hypertrophied astrocytes with thinner processes were evident. Only a few CD68 positive microglia were observed in JNCL cases (**c**), compared to the relative abundance of activated CD68 immunoreactive microglia activation present in the cortex of LINCL cases. Scale bars = 120 μm (**a**, **b**) 20 μm in higher magnification views; 50 μm (**c**). Roman numerals in **a**, **b** indicate cortical laminar boundaries

We compared the extent of gliosis in the primary visual cortex (V1) of *Cln3* ^*−/−*^ mice, (from 6.5–21 months of age) with that of wildtype controls and *Tpp-1* ^*−/−*^ mice (at 4 months of age, representing disease end stage), a model for CLN2 disease [84], an earlier onset and more rapidly progressing type of NCL [4]. In V1 of these severely affected *Tpp-1* ^*−/−*^ mice*,* the morphological features characteristic of reactive astrocytosis were evident within these astrocytes, with intense GFAP immunoreactivity, thickened processes and pronounced hypertrophy (Fig. 1a). This astrocytosis in *Tpp-1* ^*−/−*^ mice displayed laminar specificity, being most pronounced in laminae II and III, V and VI.

In comparison, in V1 of *Cln3* ^*−/−*^ mice, GFAP immunoreactivity revealed a markedly different extent of astrocytosis, with substantial differences in astrocyte morphology. Apart from a small population of darkly immunostained astrocytes present in the most ventral portion of lamina VI, the majority of astrocytes in V1 of presymptomatic 6.5 month old *Cln3* ^*−/−*^ mice displayed a protoplasmic morphology with numerous thin processes, compared to the characteristic appearance of fully activated astrocytes in *Tpp-1* ^*−/−*^ mice (Fig. 1a). Initially largely confined to laminae I and IV, these protoplasmic astrocytes became more widespread within V1 of *Cln3* ^*−/−*^ mice with disease progression, being present in lamina II and most of laminae V and VI at 12 months of age, and additionally in lamina IV by 21 months of age. Although the intensity of GFAP immunoreactivity progressively increased with time, only a small proportion of these astrocytes (mostly within lamina VI) displayed thickened processes or an enlarged cell soma, with most retaining a protoplasmic appearance with thin processes, even in severely affected 21 month old *Cln3* ^*−/−*^ mice. These data suggest that at least a subset of *Cln3* ^*−/−*^ astrocytes do retain the ability to transform morphologically, but only in a protracted manner towards disease end-stage.

A similarly attenuated activation of microglia was also evident in the cortex of *Cln3* ^*−/−*^ mice (Fig. 1b). Although CD68 immunoreactive microglia were clearly more darkly immunostained in V1 of *Cln3* ^*−/−*^ mice than in WT controls at all ages examined, these microglia retained a relatively small cell soma and numerous long thin processes. Only in severely affected *Cln3* ^*−/−*^ mice by 21 months, were more overtly swollen and activated microglia were seen, and these were largely restricted to the more ventral portion of lamina VI and V. Even then, many of these swollen CD68 positive cells retained long thickened processes. This is in marked contrast to the V1 of 4-month-old *Tpp-1* ^*−/−*^ mice that were at disease end-stage, where numerous intensely immunostained, completely rounded, and fully activated amoeboid microglia were evident.

Taken together, these data suggest that loss of *Cln3* impairs the morphological transformation of both astrocytes and microglia, which is limited and only occurs late in the disease, in more severely affected *Cln3* ^*−/−*^ mice. Having described a relatively limited glial response in the hippocampus of human CLN3 disease cases [90], we next investigated whether a similar phenotype was also evident in the primary visual cortex of CLN3 disease patient brain autopsy material (Fig. 1c). Numerous intensely immunostained GFAP positive hypertrophied astrocytes with many thickened processes were observed in V1 of CLN2 cases. In contrast, only a few weakly immunostained GFAP expressing astrocytes with thin processes were present in this region of CLN3 disease cases (Fig. 1c, top row). Similarly, dramatically fewer CD68 positive microglia were observed in V1 of CLN3 disease cases compared with CLN2 disease cases (Fig. 1c, bottom row).

These morphological observations suggest that the basic biology of glia, the neuronal support cells, may be impaired in CLN3 disease. To begin investigating how loss of CLN3 expression could influence glial cell function, we compared the properties of *Cln3* ^−/−^ and WT glial cells using primary cultures of either astrocytes or microglia, having first defined the composition of our astrocyte (Additional file 1: Figure S1 and Additional file 2: Figure S2) and microglial monocultures (Additional file 3: Figure S3). One week after plating microglial cultures showed over 99% of DAPI stained cells expressed CD68 (99.97 ± 0.02% and 99.97 ± 0.02% CD68 + ve in WT and *Cln3* ^−/−^, respectively; with only 0.03 ± 0.02% and 0.03 ± 0.03% being GFAP + ve). One week after plating over 98% of DAPI positive cells in our astrocyte cultures were positive for GFAP (WT: 98.80 ± 0.28% GFAP + ve, 1.90 ± 0.17% CD68 + ve, and 0.10 ± 0.10% O4 + ve; *Cln3* ^−/−^: 98.86 ± 0.10% GFAP + ve, 1.05 ± 0.13% CD68 + ve, and 0.10 ± 0.03% O4 + ve). With subsequent time in culture an increased fraction of DAPI stained, but GFAP negative cells were apparent in our astrocyte cultures, especially those from WT mice (e.g. see Fig. 3A.). However, at these later time points virtually all these DAPI labelled cells immunostained positively for a second astrocyte marker glutamine synthetase (see Additional file 2: Figure S2 for an example taken 48 h later), suggesting a dynamic down-regulation of GFAP over time under some culture conditions. Nevertheless, a minor contamination of our astrocyte cultures with ependymal or endothelial cells cannot be excluded.

### Morphological responses of *Cln3*^*−/−*^ glia to activation are attenuated

To determine whether the attenuated morphological response of *Cln3* ^*−/−*^ glia observed in vivo was mimicked in vitro*,* we stimulated monocultures of either microglia and astrocytes and assessed their ability to undergo morphological changes. To do this we exposed cultures to standard inflammatory stimuli that up-regulate pathways associated with immune and injury-related functions [38], either the bacterial endotoxin lipopolysaccharide (LPS) alone to activate microglia, or to LPS combined with interferon-γ (IFN), which synergizes with the effects of LPS, to activate astrocytes [26].

**Fig. 2:**
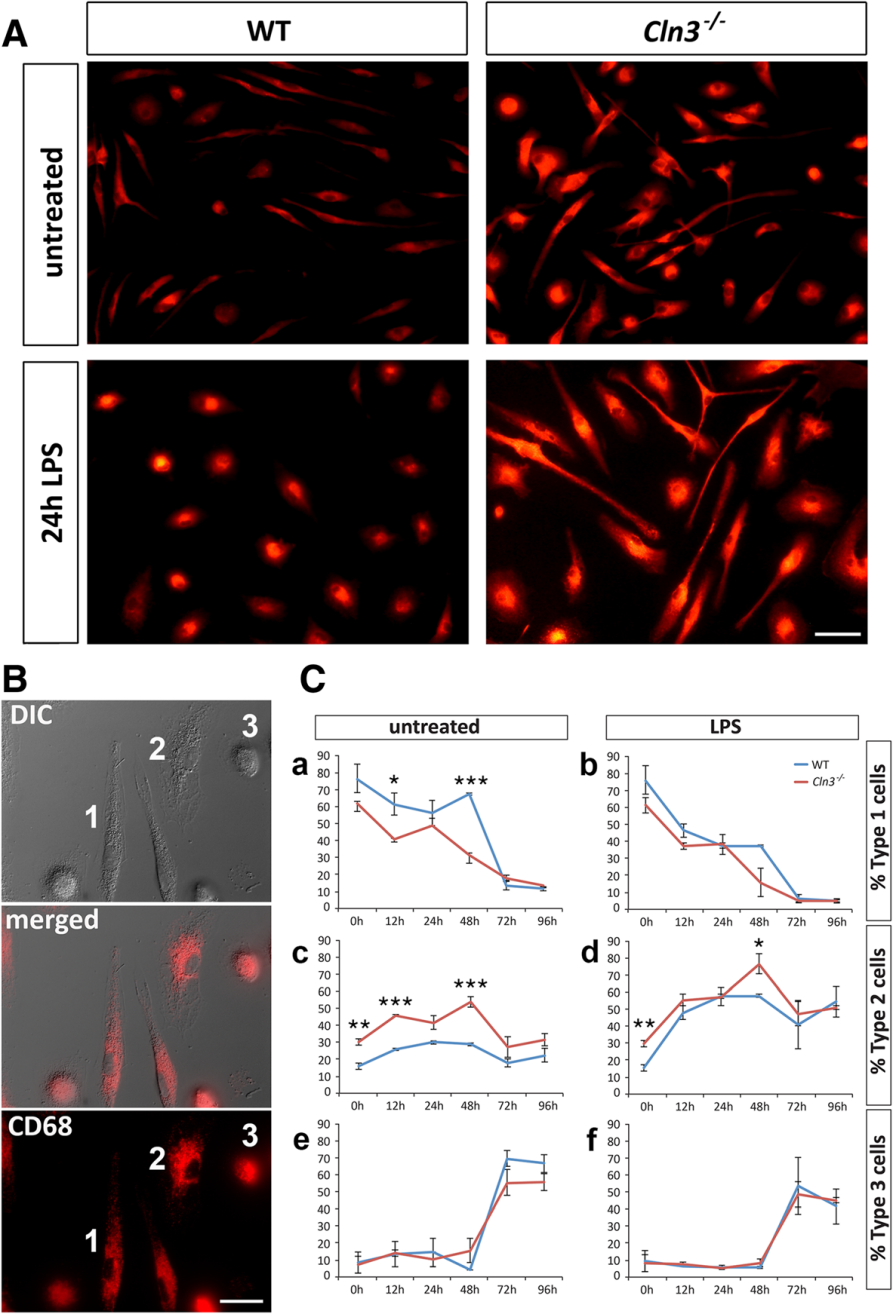
Attenuated morphological transformation of *Cln3* ^*−/−*^ microglia. The morphology of wild type (WT) and *Cln3*-deficient (*Cln3* ^*−/−*^) microglia studied under basal conditions and after stimulation with LPS was revealed by CD68 immunostaining (*red*). **A** Cultures of unstimulated WT microglia were mainly bipolar but upon LPS stimulation for 24 h these cells rapidly changed shape. In contrast, while cultures of *Cln3* ^*−/−*^ microglia exhibited a heterogeneous morphology and had intense immunostaining for CD68 under basal conditions these cells failed to dramatically change shape upon stimulation for 24 h. **B** To quantify morphological changes over time, cells were divided into three categories: Type 1 cells (resting microglia); Type 2 cells (migrating/activated microglia); type 3 (amoeboid activated microglia). **C** The transformation of type 1 cells into type 2 cells initially occurred more slowly in *Cln3* ^*−/−*^ microglial cultures upon stimulation, with type 3 cells first appearing in cultures of both genotypes around 48 h regardless of treatment. Scale bars = 50 μm (**A**, **B**)

We first confirmed that *Cln3* ^*−/−*^ glia were able to activate relevant downstream signaling pathways (phosphorylated NF-κβ subunit p65 and phosphorylated STAT-1, as downstream effectors of LPS and IFNγ stimulation respectively, [22, 94]) (Additional file 4: Figure S4). Next, to characterize the morphological transformation of microglia, WT and *Cln3* ^*−/−*^ microglial cultures were immunostained with CD68 at various time-points after activation (2, 6, 12, 24, 48, 72 and 96 h), dividing these cells according to their morphology into Type 1 (non-activated), Type 2 cells (partly activated) and Type 3 cells (fully activated) (see methods). Even under basal conditions, CD68 immunoreactivity appeared more intense in *Cln3* ^*−/−*^ vs. WT microglial cultures, with more rounded cells present (Fig. 2A). Upon stimulation, microglia of both genotypes morphologically transformed, but many fewer *Cln3* ^*−/−*^ microglia changed shape after 24 h (Fig. 2A). When these changes were quantified (see Fig. 2B), there were more type 2 cells in *Cln3* ^*−/−*^ vs. WT microglial cultures under basal conditions (Fig. 2C, compare panels a and b), suggesting a higher level of basal activation, but a slower morphological transformation of CLN3 disease microglia. A transformation of Type 1 cells into Type 2 cells occurred in microglial cultures of both genotypes upon stimulation, however, *Cln3* ^*−/−*^ microglia responded more slowly than WT microglia, with a slower decline in the number of Type 1 cells (Fig. 2C, a, b) and a slower increase in the number of Type 2 cells (Fig. 2C, compare panels c and d). Until 48 h very little change was observed in the percentage of Type 3 cells under any condition (Fig. 2C), but by 72 h there was a dramatic increase in the proportion of this fully activated cell type within both WT and *Cln3* ^*−/−*^ microglial cultures under all conditions (Fig. 2c–f). This change was accompanied by a reduction in the percentage of both Type 1 and Type 2, suggesting a morphological transformation into Type 3 cells with increased time in culture.

**Fig. 3:**
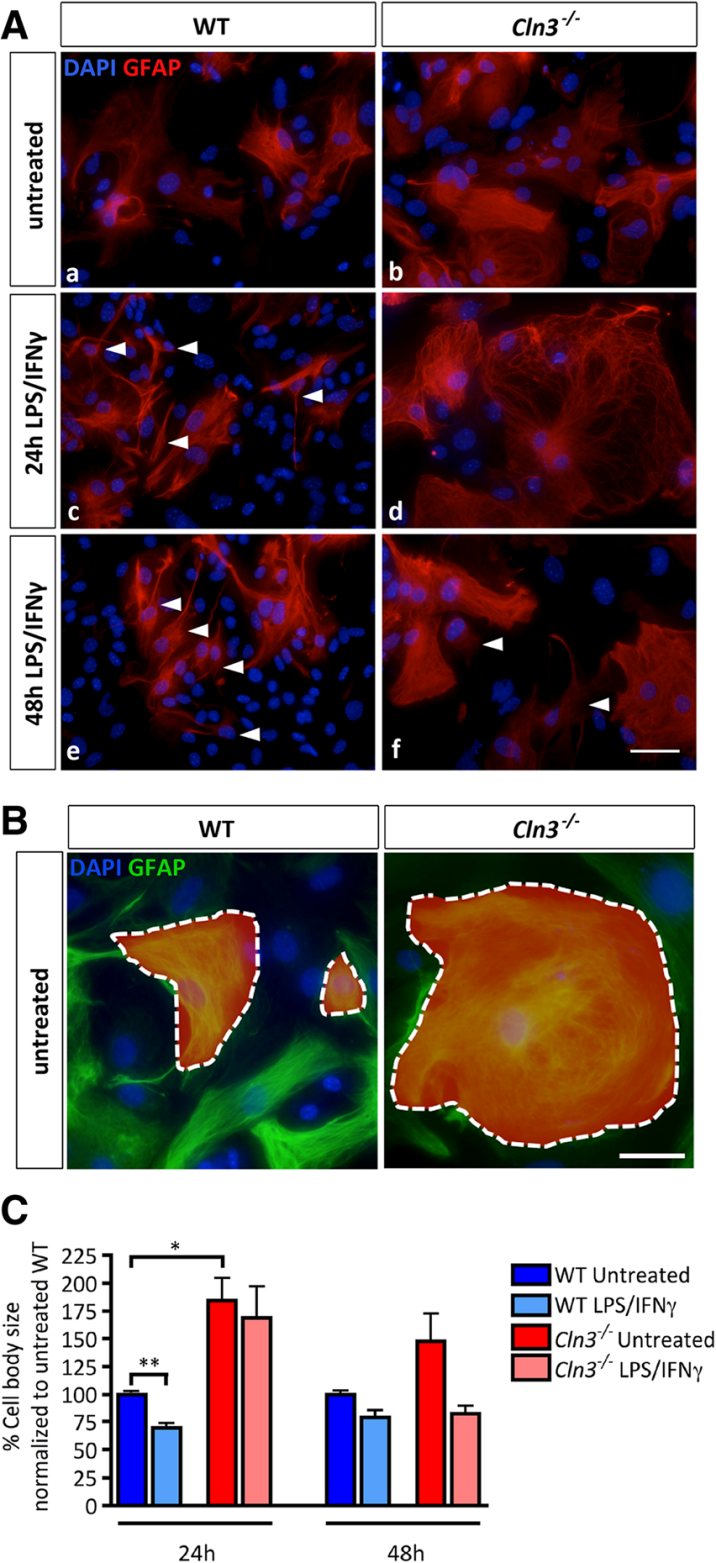
Attenuated morphological transformation of *Cln3* ^*−/*−^ astrocytes. The morphology of wild type (WT) and *Cln3*-deficient (*Cln3* ^*−/−*^) astrocytes was studied under basal conditions and after stimulation with LPS/IFNγ for 24 or 48 h by immunostaining with GFAP (*red* in **A**, *green* in **B**). DAPI (*blue*) was used to visualize all nuclei. **A** WT astrocytes changed their morphology dramatically after a 24 h exposure to LPS/INFγ to display characteristic branched processes (arrowheads) and these changes became enhanced over time. In contrast *Cln3* ^*−/−*^ astrocytes remained relatively morphologically unchanged after 24 h of stimulation remaining as large flat cells with no processes, but showed morphological changes after 48 h activation. **B** *ImageJ* was used to quantify astrocyte cell body size under all experimental conditions by drawing around the soma of GFAP positive cells (*dashed lines*, with contained area *shaded red*). **C** The mean cell soma sizes were determined by quantifying 10 random fields per coverslip and a minimum of two coverslips per experiment. Scale bar = 50 μm

The morphological response of astrocytes to stimulation (LPS/INFγ treatment for 24 h or 48 h) was assessed in GFAP immunostained cultures. Even under basal conditions, untreated *Cln3* ^*−/−*^ astrocytes had a strikingly different morphology to WT astrocytes, appearing larger and flatter, with disrupted intermediate filaments (Fig. 3). Upon stimulation, WT astrocytes already began to morphologically transform after 24 h; changing from broad, non-process bearing, flat cells into cells with a shrunken soma and multiple branched processes (as described in [53]) (Fig. 3A, c arrowheads). These changes become more apparent with time (Fig. 3A, e). In contrast, no significant morphological transformation of *Cln3* ^*−/−*^ astrocytes could be detected until 48 h stimulation, when soma size began to decrease and some cells developed processes (Fig. 3A, f). To quantify these changes the soma size of WT and *Cln3* ^*−/−*^ astrocytes were compared (Fig. 3B). After activation for 24 h or 48 h, the cell soma of WT astrocytes became smaller, and this was statistically significant after 24 h (30.5% ± 3.3 decrease). After 24 h of stimulation the soma size of *Cln3* ^*−/−*^ astrocytes remained unchanged, but after 48 h of stimulation was not statistically different to that of stimulated WT astrocytes (Fig. 3C).

These data demonstrate that *Cln3* ^*−/−*^ astrocytes and microglia are attenuated in their ability to change their morphology upon stimulation, suggesting that these cells retain at least some of their in vivo disease characteristics when cultured.

### *Cln3*^*−/−*^ astrocytes, but not *Cln3*^*−/−*^ microglia, have a disrupted cytoskeleton

Since morphological changes require cytoskeletal rearrangements, and GFAP immunostaining suggested that intermediate filament organization was perturbed in *Cln3* ^*−/−*^ astrocytes (Fig. 3A), we also immunostained astrocytes for α- and β-tubulin to visualize microtubules and with phalloidin to visualize F-actin filaments and a similar cytoskeletal analysis was performed with microglia.

**Fig. 4:**
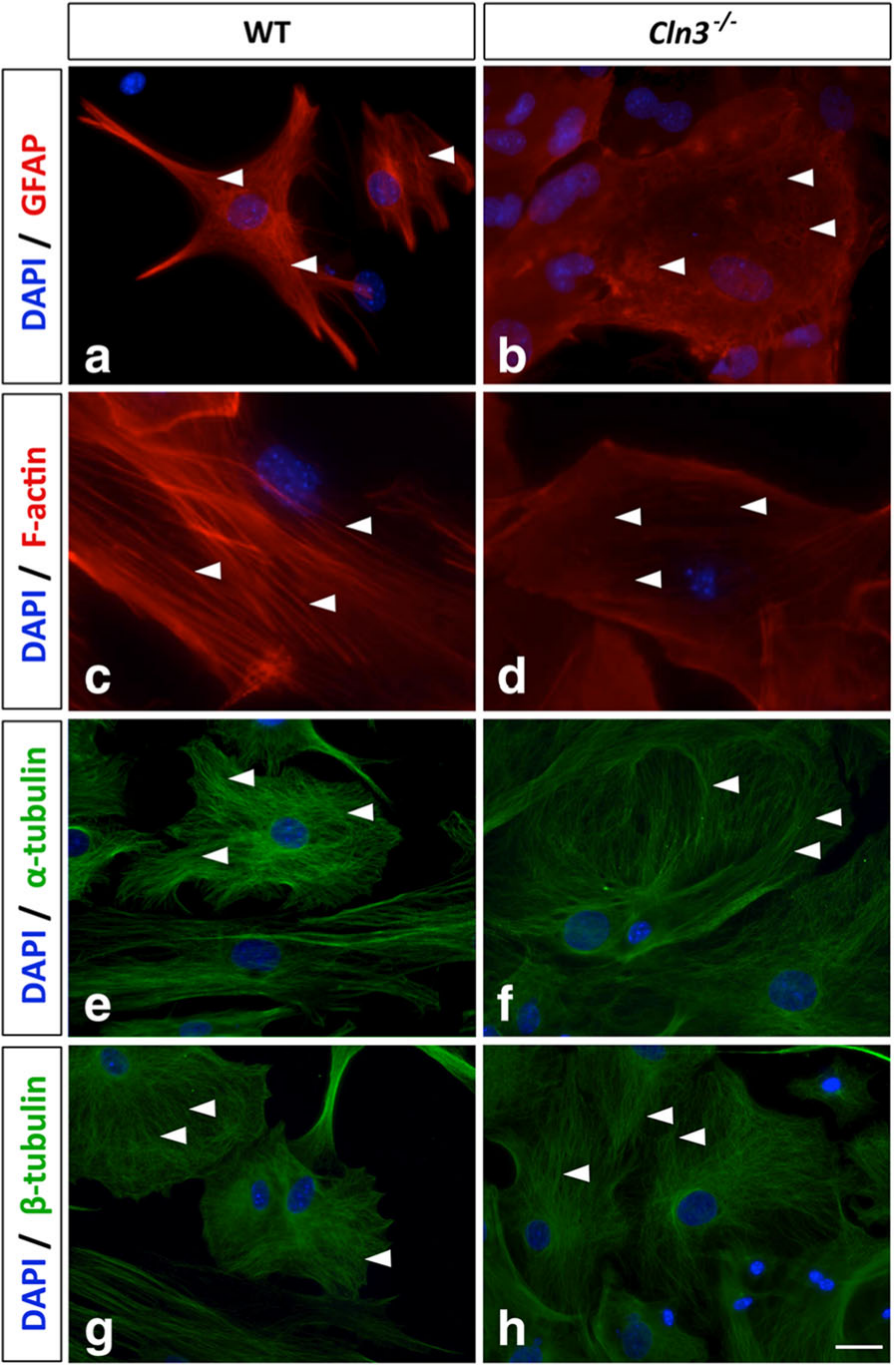
*Cln3* ^*−/−*^ astrocytes have a disrupted cytoskeleton. The cytoskeletal organization of wild type (WT) and *Cln3*-deficient (*Cln3* ^*−/−*^) astrocytes was determined by immunostaining with GFAP (*red* in **a**, **b**) to visualize intermediate filaments, phalloidin to visualize F-actin (*red* in **c**, **d**), and α- or β-tubulin to visualize microtubules (*green* in **e**, **f** and in **g**, **h**, respectively). DAPI was used to visualize nuclei (*blue*). WT astrocytes had a well-organized intermediate filament, F-actin and microtubule cytoskeleton (arrowheads in **a**, **c**, **e**, **g**), while *Cln3* ^*−/−*^ astrocytes had a highly disrupted intermediate filament and F-actin cytoskeleton (arrowheads in **b** and **d**) but their microtubule structure appeared normal (arrowheads in **f** and **h**). The cell body size of *Cln3* ^*−/−*^ astrocytes appeared larger than that of WT astrocytes. Scale bar = 25 μm

Both intermediate filaments and F-actin filaments appeared less defined and highly disorganized in *Cln3* ^*−/−*^ vs. WT astrocytes (Fig. 4, a, b for GFAP, and c, d for F-actin, examples marked with arrowheads). Most *Cln3* ^*−/−*^ astrocytes lacked F-actin filaments that spanned the cell body, a common morphological feature of cultured WT astrocytes (Fig. 4c, d). However, the α- and β-microtubular organization of WT and *Cln3* ^*−/−*^ astrocytes appeared similar (Fig. 4, e, f and g, h, examples marked with arrows). These data reveal that enlarged *Cln3* ^*−/−*^astrocytes have an abnormally organized actin and intermediate filament cytoskeleton, whilst their microtubule organization appears normal. No overt changes were observed in the cytoskeletal organization of *Cln3* ^*−/−*^microglia (data not shown).

### *Cln3*^*−/−*^ glia show altered protein secretion profiles

The secretion of soluble factors is a key feature of both microglia and astrocytes under both physiological and pathological conditions [51], therefore we compared the secretion profiles of WT and *Cln3* ^*−/−*^ glia under basal conditions and after stimulation.

**Fig. 5:**
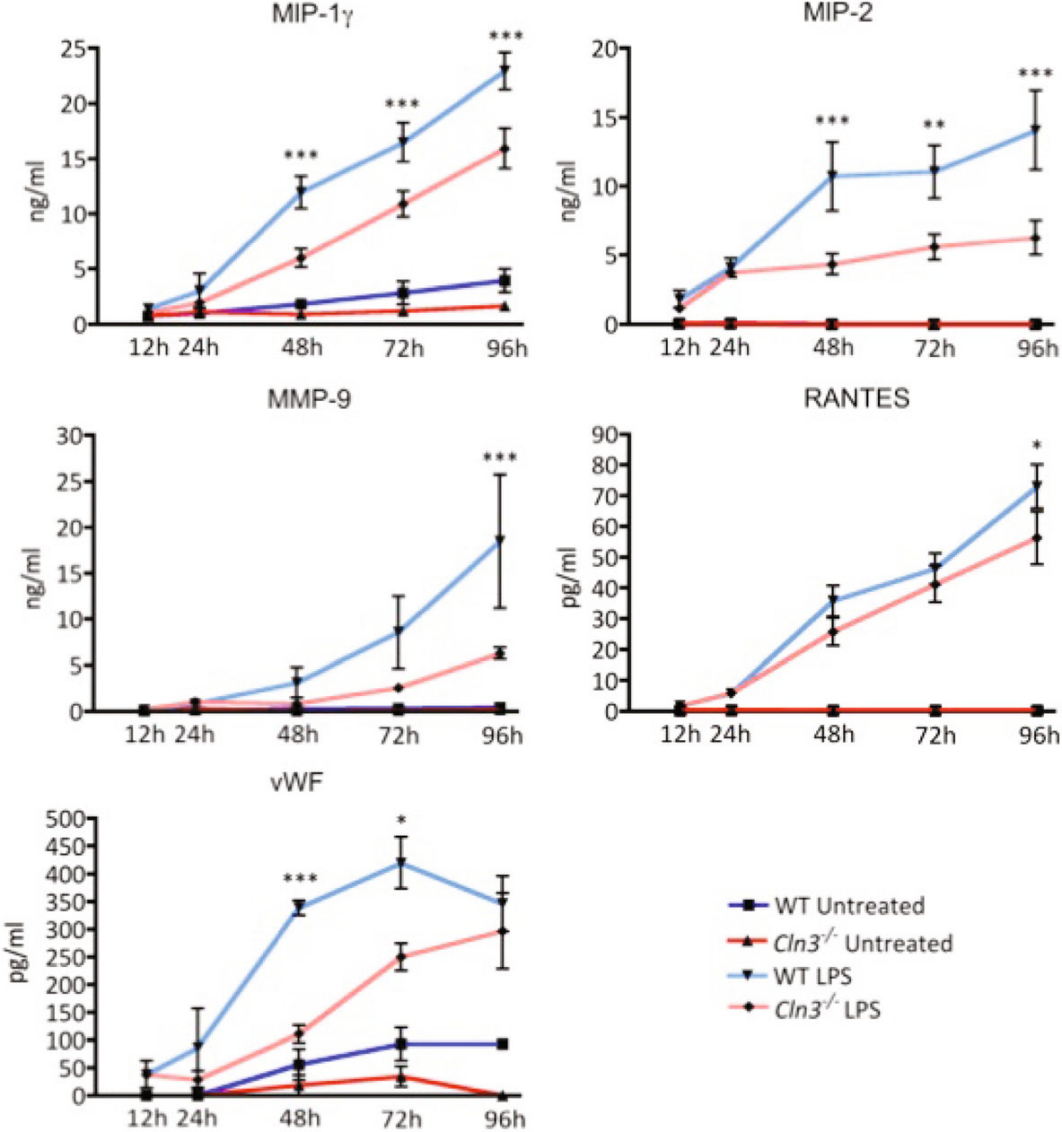
Altered protein secretion profiles of *Cln3* ^*−/−*^ microglia. Secreted protein levels were quantified from supernatants collected from WT and *Cln3* ^*−/−*^ microglial cultures grown under basal conditions, or after stimulation with LPS. Following quantitative analysis of 59 soluble factors, 5 were found to be secreted at significantly lower levels by *Cln3*-deficient (*Cln3* ^*−/−*^) microglia over time in culture compared to wild type (WT) microglial cultures. These included the chemokines MIP-1-γ, MIP-2 and RANTES, the glycoprotein vWF, and the matrix metalloproteinase MMP-9

*Cln3* ^*−/−*^ and WT microglia did not display any differences in the levels of factors secreted under basal conditions (data not shown), but five proteins were secreted at significantly lower levels by *Cln3* ^*−/−*^ microglia upon LPS stimulation (Fig. 5). These proteins included three chemokines (MIP-1-γ, MIP-2 and RANTES), a glycoprotein (vWF) and a matrix metalloproteinase (MMP-9). These data indicate that *Cln3* ^*−/−*^ microglia retain their capacity to secrete the majority of soluble factors into their environment. Equally, we observed no difference in the phagocytic properties of *Cln3* ^*−/−*^ microglia grown under basal conditions or after stimulation (data not shown).

Under basal conditions there was no significant difference between WT and *Cln3* ^*−/−*^ astrocytes in the secretion pattern of the majority of proteins analyzed (Additional file 5: Table S1). However, upon exposure to LPS/IFN-γ a broad range of secreted proteins were detected at significantly reduced levels in *Cln3* ^*−/−*^ astrocyte supernatants (Additional file 5: Table S2). Indeed, out of the 59 screened factors none (at 6 h), 19 (at 24 h) and 42 (at 72 h) factors were secreted at significantly lower levels by *Cln3* ^*−/−*^ astrocytes compared to WT astrocytes upon activation (Additional file 5: Table S1 and S2). These included the reduced secretion of several mitogens (M-CSF, IL-3, FGF2, GM-CSF, FGF-9, TPO and IL-5), chemokines (Eotaxin, MIP1α, MCP-3, MCP-1, KC/GROα, MIP-3α, MIP-2, IP-10, RANTES, MDC, MCP-5, MIP-1γ, GCP-2 and MIP-1β), anti- and pro-inflammatory cytokines (IL-17α, IL-6, IL-12p70, IL-1α, TNFα, IL-1β, IL-10, IL-2). Furthermore, these cells also showed an impaired ability to secrete a range of proteins shown to have neuroprotective properties: IL-6 [95], IL-3 [101], GM-CSF [55], IL-10 [7], LIF [57], MCP-1 and RANTES [15].

**Fig. 6:**
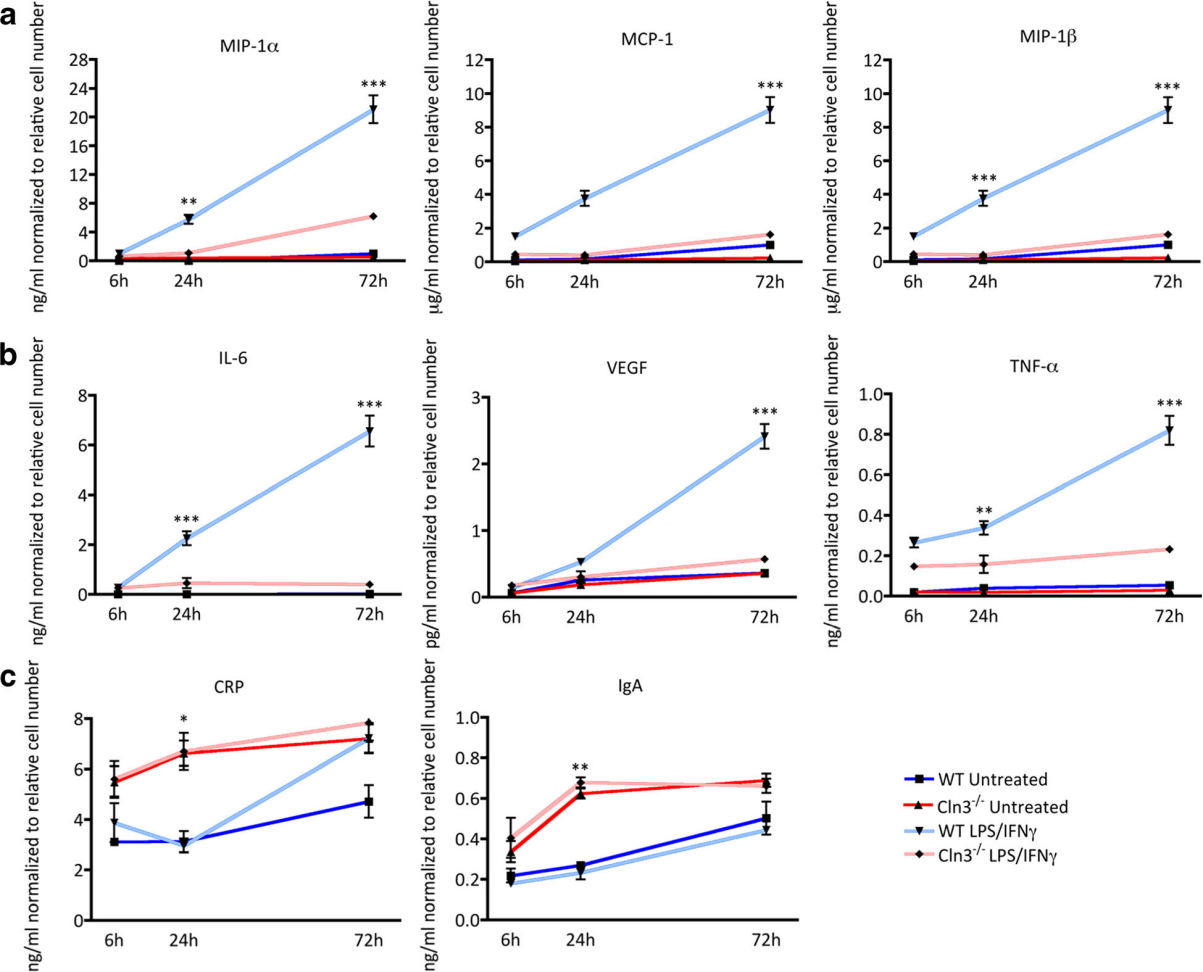
*Cln3* ^*−/−*^ astrocytes show differences in their ability to secrete proteins. Secreted protein levels were quantified from supernatants collected from wild type (WT) and *Cln3*-deficient (*Cln3* ^*−/−*^) astrocyte cultures grown under basal conditions or after stimulation with LPS/IFNγ. Examples of chemokines (**a**), neuroprotective factors (**b**) and proteins secreted at elevated levels by *Cln3* ^*−/−*^ astrocytes following activation (**c**), are shown. **a** *Cln3* ^*−/−*^ astrocytes secreted significantly less MIP-1ß, MIP-1α and MCP-1 when treated with LPS/IFNγ than did WT astrocytes. There was no significant difference between untreated WT and *Cln3* ^*−/−*^ samples. **b** *Cln3* ^*−/−*^ astrocytes secreted significantly less IL-6, TNF-α and VEGF upon activation than did *WT astrocytes*. There was no significant difference between untreated samples. **c** *Cln3* ^*−/−*^ astrocytes secreted significantly more CRP and IgA after 24 h exposure to LPS/IFNγ, no such change was observed in WT astrocytes

There were however exceptions to this pattern of altered protein secretion. Two chemokines, macrophage inflammatory protein-1γ (MIP-1γ) and granulocyte chemotactic protein 2 (GCP-2) were both secreted significantly less by *Cln3* ^*−/−*^ astrocytes under basal conditions, while one protein, tissue factor (TF), was secreted more by *Cln3* ^*−/−*^ astrocytes under basal conditions. In contrast fibrinogen and the mitogen CRP were secreted significantly more by *Cln3* ^*−/−*^ astrocytes upon stimulation. Examples of astrocyte secretion profiles for specific proteins are shown in Fig. 6.

### Altered glutathione handling by *Cln3*^*−/−*^ astrocytes

The altered secretion profile of *Cln3* ^*−/−*^ astrocytes led us to consider that these cells may also fail in their antioxidant support of neurons, particularly in their ability to secrete glutathione (GSH), one of the brain’s most important neuroprotective factors [31, 78].

**Fig. 7:**
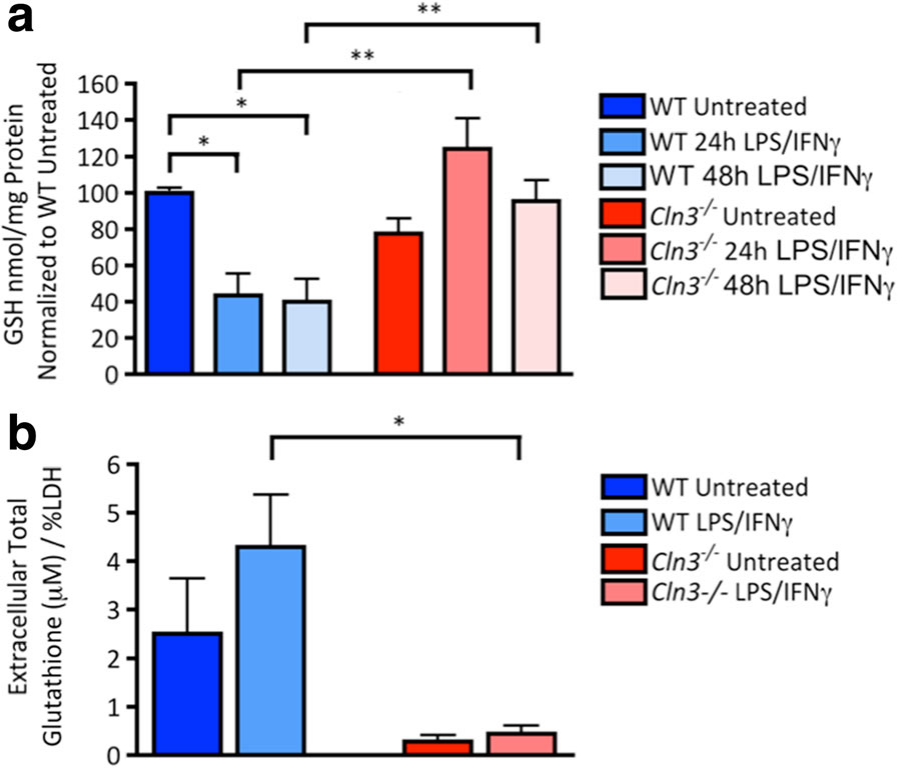
*Cln3* ^*−/−*^ astrocytes fail to secrete glutathione. Wild type (WT) and *Cln3*-deficient (*Cln3* ^*−/−*^) primary cortical astrocytes were analyzed for their ability to synthesize and secrete reduced glutathione (GSH). **a** Reverse phase HPLC was used to measure the intracellular levels of GSH in WT and *Cln3* ^*−/−*^ astrocytes treated with LPS/IFNγ for 24 or 48 h, or from untreated samples. The GSH levels in each sample were normalized to the total amount of protein in that sample and results presented in nmol/mg of protein. Additionally, these results were normalized to untreated WT astrocyte GSH levels in each experiment. LPS/IFNγ stimulation caused a significant decrease in the intracellular levels of GSH in WT astrocytes but not in *Cln3* ^−/−^ astrocytes. **b** The GSH-Glo kit was used to measure the total amount of GSH secreted into the medium over an 8 h period by cultures of untreated and LPS/IFNγ treated WT and *Cln3* ^*−/−*^ astrocytes. TCEP (12uM) was used to convert the oxidised form of glutathione (GSSH) to GSH to measure the total amount of GSH in each sample. These results were normalized to released %LDH from total LDH. LPS/IFNγ treated *Cln3* ^*−/−*^ astrocytes secreted significantly reduced levels of GSH compared to LPS/IFNγ treated WT astrocytes

To analyze the ability of astrocytes to make and secrete GSH, cultures were washed, lysed and the intracellular levels of the reduced (functional) form of GSH measured. Under basal conditions, no significant differences in GSH levels were detected between WT and *Cln3* ^*−/−*^ cultures (Fig. 7a). However, following stimulation the intracellular levels of reduced GSH decreased significantly in WT cultures (56.3 ± 12.0% reduction after 24 h exposure, 59.9 ± 12.6% reduction after 48 h, Fig. 7a), but were markedly increased in *Cln3* ^*−/−*^ cultures (63 ± 33.6% increase after 24 h, 28.1 ± 19.3% increase after 48 h, Fig. 7a). Thus, intracellular GSH levels in LPS/IFNγ treated WT astrocytes were significantly lower than in LPS/IFNγ treated *Cln3* ^*−/−*^ astrocytes after 24 (59.6 ± 12.2% decrease in WT vs. *Cln3* ^*−/−*^) and 48 h (51.9 ± 14.3% decrease in WT vs. *Cln3* ^*−/−*^). These data suggest a failure of *Cln3* ^*−/−*^ astrocytes to secrete GSH, consistent with the observation that significantly higher levels of extracellular GSH could be detected in the medium from WT vs. *Cln3* ^*−/−*^ astrocytes after stimulation (Fig. 7b).

Since the method described above only measures the reduced form of glutathione, it is conceivable that these results could be explained by an opposing change in the level of oxidized glutathione (GSSG). This possibility was excluded by indirectly measuring intracellular GSSG levels by converting GSSG to GSH using glutathione reductase (GR). Subsequent HPLC analysis revealed no measurable difference between the intracellular levels of GSH and total glutathione (after GR treatment) in any of these samples, suggesting that all the intracellular glutathione in both WT and *Cln3* ^*−/−*^ astrocytes is present in the reduced form (data not shown).

The actin cytoskeleton is important for exocytosis in astrocytes [70], and it appears abnormally organized in *Cln3* ^*−/−*^ astrocytes (Fig. 3A). Therefore, the possible connection between the disrupted actin cytoskeleton and impaired glutathione secretion was examined by treating WT astrocytes with cytochalasin D (1uM for 30 min before the 8 h measurement period) to inhibit the polymerization of actin. This resulted in a significant reduction (50.1 ± 12.7%) in the levels of extracellular glutathione in LPS/IFNγ treated WT astrocytes (Additional file 6: Figure S5). Thus, a normal actin cytoskeleton is essential for glutathione secretion by astrocytes.

Since the defects in *Cln3* ^*−/−*^ astrocytes appeared more profound than those in microglial cells, and the cytoskeletal disruption observed in these cells could impact many of their functions, we investigated whether *Cln3* ^*−/−*^ astrocytes could perform other key tasks effectively.

### An impaired ability of *Cln3*^*−/−*^ astrocytes to migrate, clear glutamate and signal via Ca^2+^

#### Slower migration of Cln3^−/−^ astrocytes

**Fig. 8:**
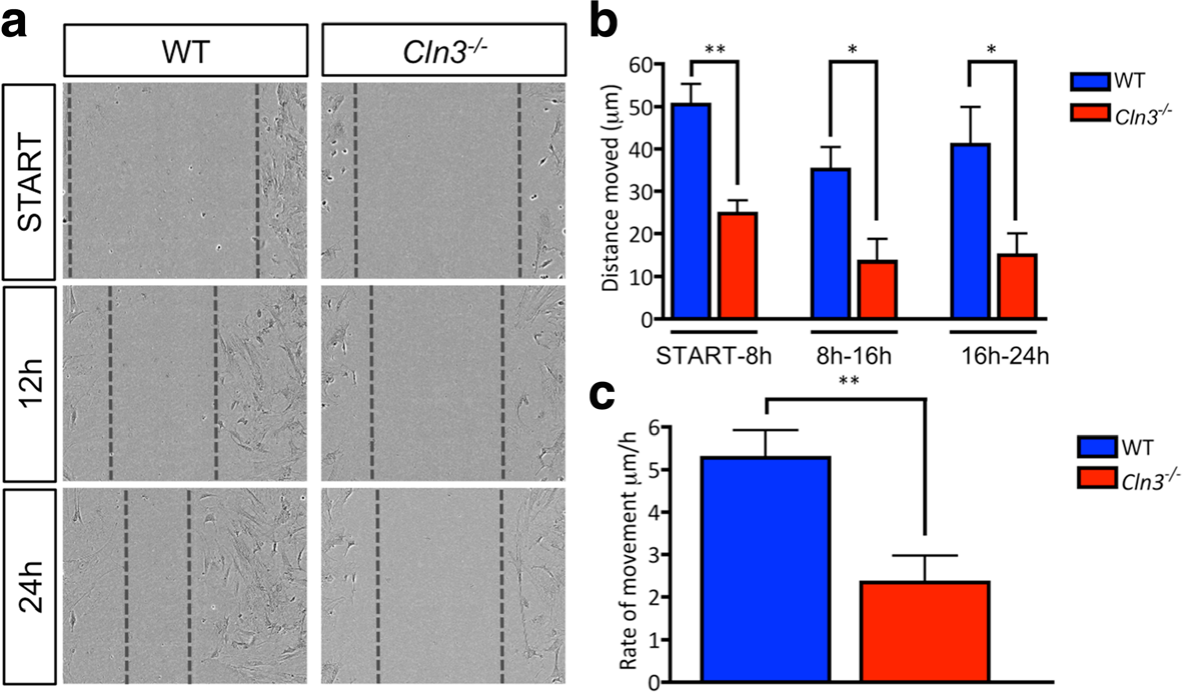
*Cln3* ^*−/−*^ Astrocytes have a migration defect. Wild type (WT) and *Cln3*-deficient (*Cln3* ^*−/−*^) primary cortical astrocytes were plated on Essen Image Lock 24 well plates, grown to confluence then scratched using an Essen wound maker. **a** Representative pictures of the wound at three time points. **b** The distance migrated by WT and *Cln3* ^*−/−*^ astrocytes every 4 h was calculated by comparing wound widths between the start and the different time points. WT astrocytes migrated significantly further than did *Cln3* ^*−/−*^ astrocytes. **c** The rate of migration was measured by calculating the distance migrated by these cells/h. *Cln3* ^*−/−*^ astrocytes migrated significantly slower than WT astrocytes. In each experiment three wound widths were measured per well and three wells quantified per experiment

Astrocyte migration is associated with local inflammation [17] and requires cytoskeletal rearrangements, raising the likelihood that this process may be impaired in *Cln3* ^*−/−*^ astrocytes. To test this possibility, an Essen Wound-maker was used to create a cell-free area in confluent cultures of WT and *Cln3* ^*−/−*^ astrocytes, and the ability of the cells to migrate and fill this space assessed. WT astrocytes migrated into the cell free area rapidly and nearly closed the gap within 24 h (Fig. 8a). The distance covered by *Cln3* ^*−/−*^ astrocytes over this same time was significantly reduced (Fig. 8b). The rate of migration was significantly decreased in the absence of CLN3 (WT 5.3 ± 0.6 μm/h vs. 2.3 ± 0.6 μm/h for *Cln3* ^*−/−*^ astrocytes) (Fig. 8c), and WT astrocytes migrated further and faster than *Cln3* ^*−/−*^ astrocytes.

#### Cln3^−/−^ astrocytes show impaired glutamate clearance

**Fig. 9:**
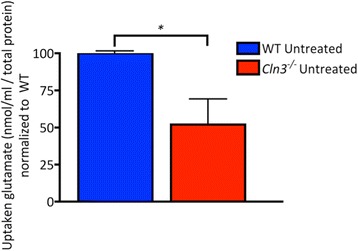
Glutamate clearance is altered in *Cln3* ^*−/−*^ astrocytes. The ability of wild type (WT) and *Cln3*-deficient (*Cln3* ^*−/−*^) astrocytes to clear glutamate from the medium was assessed using a Glutamate Assay Kit. WT and *Cln3* ^*−/−*^ astrocytes were incubated with 2 mM glutamate for 2 h, and wells without astrocytes were used as controls. The glutamate remaining in the medium was quantified and normalized to the total amount of protein, and the glutamate uptake values of *Cln3* ^*−/−*^ astrocytes were normalized to those of WT astrocyte samples. *Cln3* ^*−/−*^ astrocytes took up significantly less glutamate than did WT astrocytes over the 2 h period

A feature of JNCL pathogenesis is an elevated level of glutamate in the brains of *Cln3* ^*−/−*^ mice [65]. A glutamate assay kit revealed that *Cln3* ^*−/−*^ astrocytes take-up significantly less glutamate from the medium than WT astrocytes (48.0% ± 14.0% reduction in glutamate uptake, Fig. 9), suggesting that *Cln3* ^*−/−*^ astrocytes may not be able to scavenge excess extracellular glutamate as effectively as WT astrocytes.

#### Cln3^−/−^ astrocytes do not form a synchronized calcium wave

**Fig. 10:**
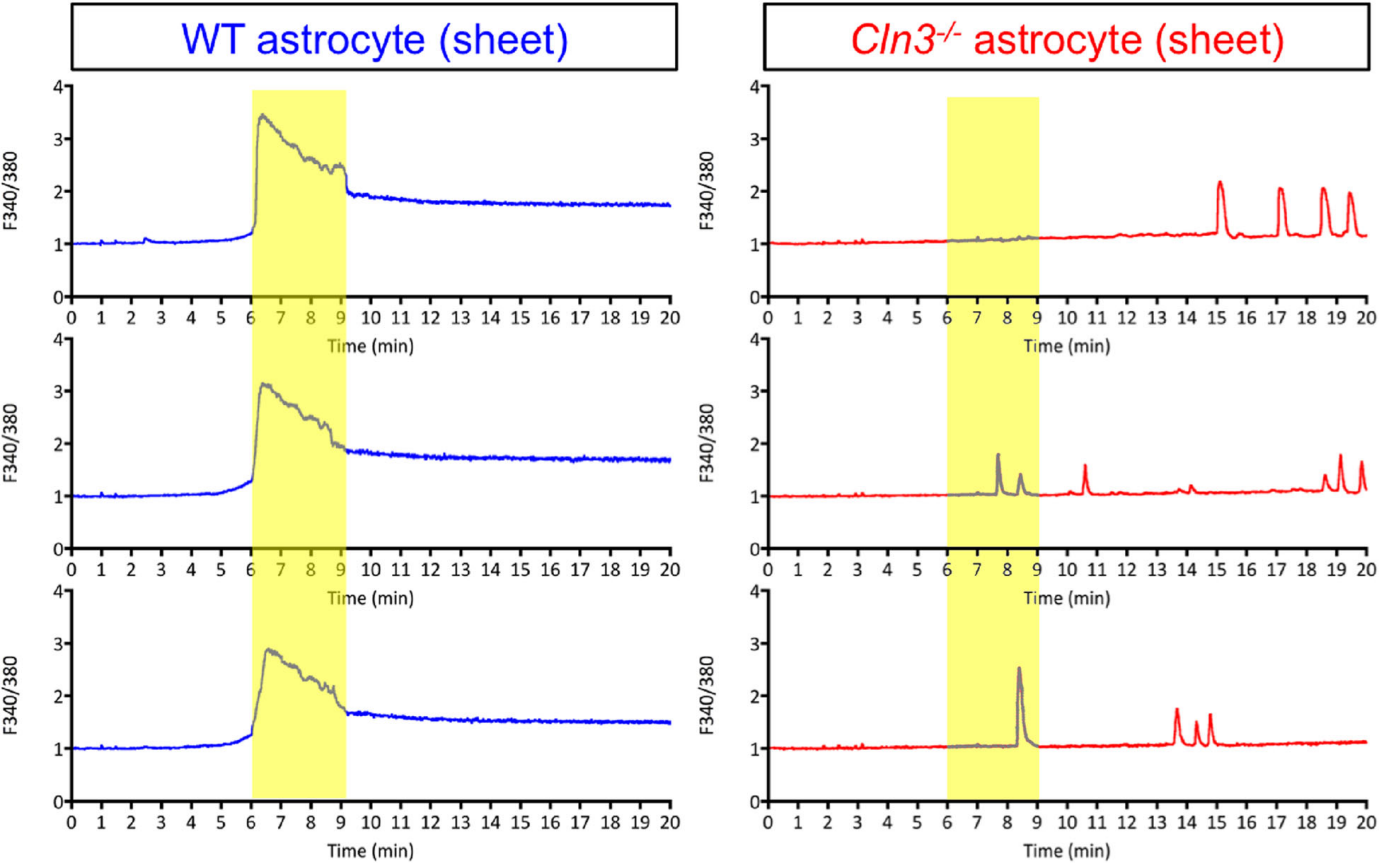
*Cln3* ^*−/−*^ Astrocytes Show Altered Calcium Signalling. Recordings of Fura-2 fluorescence were made from high density, sheet forming cultures of wild type (WT) and *Cln3*-deficient (*Cln3* ^*−/−*^) astrocytes grown under basal conditions over a period of 30-45 min, from which the first 20 min are shown. The figure illustrates changes in [Ca^2+^]_I_ in three randomly selected WT and *Cln3* ^*−/−*^ astrocytes. In response to treatment with 100 μM ATP, a propagating [Ca^2+^]_I_ wave was generated by WT astrocytes (marked with yellow bar). This synchronized [Ca^2+^]_I_ wave had a large amplitude, and a prolonged plateau persisting for several minutes after initiation. The *Cln3* ^*−/−*^ astrocytes did not exhibit any propagating calcium waves, instead, *Cln3* ^*−/−*^ astrocytes had non-synchronized, spontaneous [Ca^2+^]_I_ elevations. Data is presented as 340 nm/380 nm ratio, which directly correlates with the change in intracellular free Ca^2+^ levels

Calcium signaling forms the basis for astrocyte-astrocyte and astrocyte-neuron communication in the CNS [103]*.* Indeed*,* Ca^2+^ is exploited by astrocytes as an intercellular signal for long distance communication through functionally connected astrocyte networks. This synchronous calcium wave is propagated via gap junctions and has the potential to coordinate neurotransmitter release at all synapses within the astrocyte syncytium [92]. To explore the ability of WT and *Cln3* ^*−/−*^ astrocytes to generate a calcium wave in response to ATP [46], these cells were plated on PDL-coated 25 mm coverslips at high density so that they formed a continuous sheet. Calcium measurements were obtained after loading cells with the ratiometric and sensitive calcium indicator, Fura-2 AM, with results obtained over the first 20 min presented (Fig. 10).

A calcium wave, which initiated approximately 6 min after the start of the recordings, was obvious in high-density WT astrocyte cultures (Fig. 10, yellow bar). This synchronized, [Ca^2+^]_I_ elevation among WT astrocytes had a large amplitude (from 150% up to nearly 250% increase compared to baseline), and a clear plateau phase indicating prolonged high intracellular calcium levels after the initiation of the wave (Fig. 10). In sheet-forming *Cln3* ^*−/−*^ astrocyte cultures, however, no such synchronized [Ca^2+^]_I_ elevation was observed (Fig. 10). Instead, these *Cln3* ^*−/−*^ astrocytes exhibited sporadic calcium oscillations that did not propagate as an intercellular calcium wave. This suggests that the communication between *Cln3* ^*−/−*^ astrocytes may be severely compromised, and this in turn could impact on the control of neurotransmission.

### Altered *Cln3*^*−/−*^ neuronal morphology

We next investigated the in vitro phenotypes of *Cln3*-deficient cortical neurons. In the absence of any overt effect on intrinsic neuronal survival this analysis initially focused upon soma size, and neurite complexity.

**Fig. 11:**
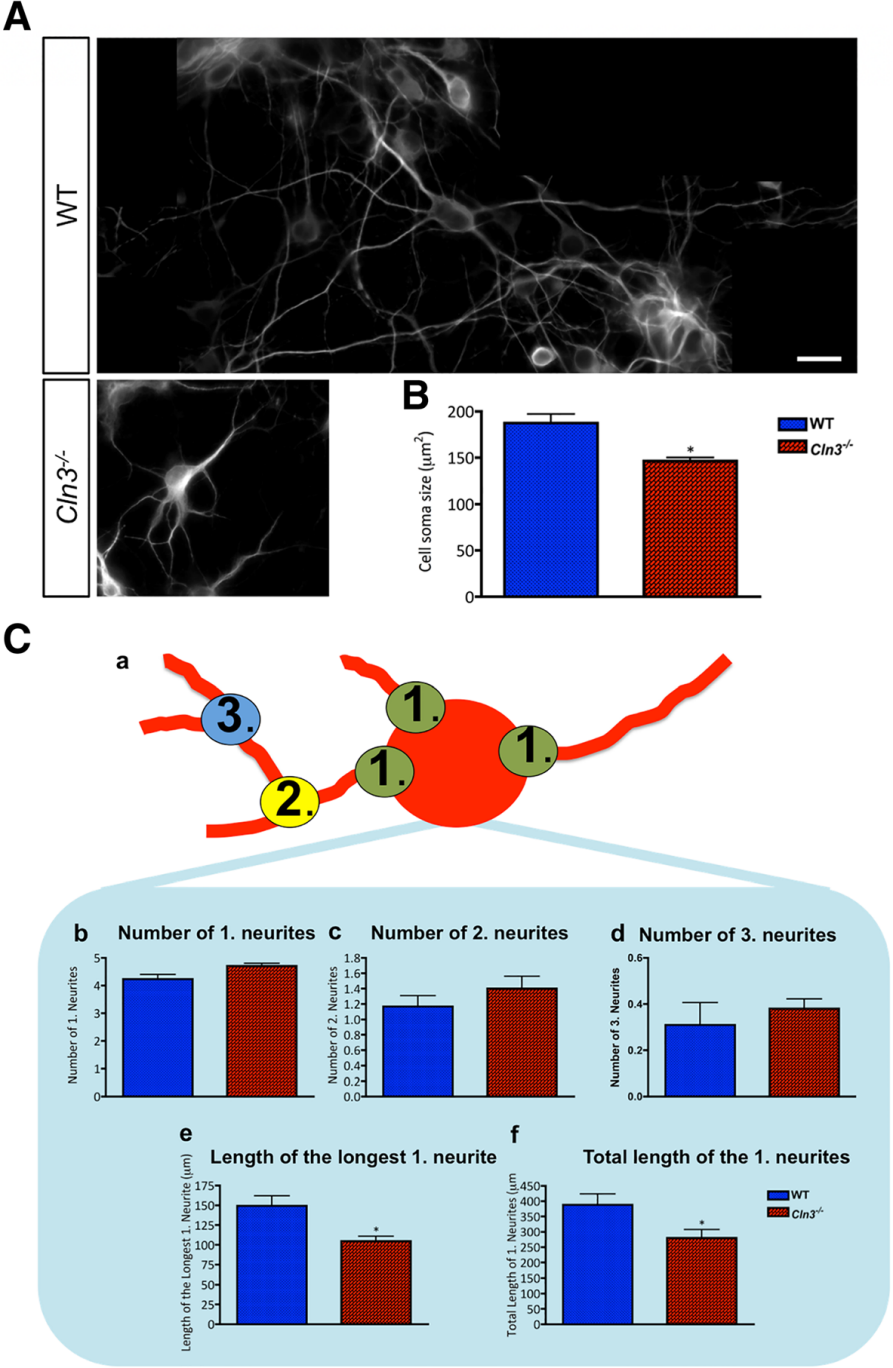
*Cln3* ^*−/−*^ cortical neurons are small and have shortened processes. The morphology of primary cortical wild type (WT) and *Cln3*-deficient (*Cln3* ^*−/−*^) neurons was compared quantitatively using ImageJ after cultures were fixed and immunostained with MAP2. **A** MAP2 expressing WT and *Cln3* ^*−/−*^ cortical neurons showing that, unlike in WT cells, MAP2 immunoreactivity is not evenly distributed between the cell soma and processes in *Cln3* ^*−/−*^ neurons. **B** Quantification of cell soma size revealed that WT neurons have a significantly bigger cell soma than *Cln3* ^*−/−*^ neurons. **C** Quantitative assessment of neurite complexity, (**a**) schematic illustration of neurite branching, showing primary neurites (1) originating directly from the cell body, secondary neurites (2) originating from primary neurites, and tertiary neurites (3) originating from secondary neurites. The length of each of the primary neurites was analyzed, and the sum of the length of all of these neurites calculated. (**b**, **c** and **d** the number of each type of neurite did not differ between WT and *Cln3* ^*−/−*^ neurons. **e** WT neurons had a longer primary neurite, and (**f**) an increased total length of the primary neurites compared to *Cln3* ^*−/−*^ neurons. Data in (**b**) and (**c**) represent mean ± SEM from approximately 40 individual cells analyzed in each experiment. This experiment was repeated three times. The scale bar in (**A**) is 20 μm

Qualitatively, the distribution of MAP2 immunoreactivity appeared different in neurons of different genotypes, appearing to be more intense within the apparently smaller cell soma of *Cln3* ^*−/−*^ neurons compared to the more even distribution within the soma and processes of WT neurons (Fig. 11A). Cell area measurements revealed *Cln3* ^*−/−*^ neuron soma to be significantly smaller than WT cells (Fig. 11B), and their neurite complexity was altered (Fig. 11C). Although *Cln3* ^*−/−*^ neurons had slightly more branching points than WT neurons, these differences were not statistically significant. However, the length of the longest primary neurite was significantly shorter in *Cln3* ^*−/−*^ neurons (Fig. 11C, e), which also displayed a significantly shorter network of primary neurites (Fig. 11C, f).

### JNCL glia are detrimental to neuronal health

The finding that *Cln3* ^*−/−*^astrocytes, and to a lesser extent microglia, have a compromised biology suggested that *Cln3* ^*−/−*^ glia could potentially have a detrimental effect on neuronal health. To test this hypothesis, primary cortical WT or *Cln3* ^*−/−*^ astrocytes and microglia were seeded on top of WT or *Cln3* ^*−/−*^ primary cortical neuronal cultures. Glia in these co-cultures were not exposed to LPS or LPS/IFNγ. As a readout of neuronal health we analyzed the same neuronal phenotypes defined above, and assessed if there were any impact upon neuronal survival, and neurite complexity in these co-cultures.

**Fig. 12:**
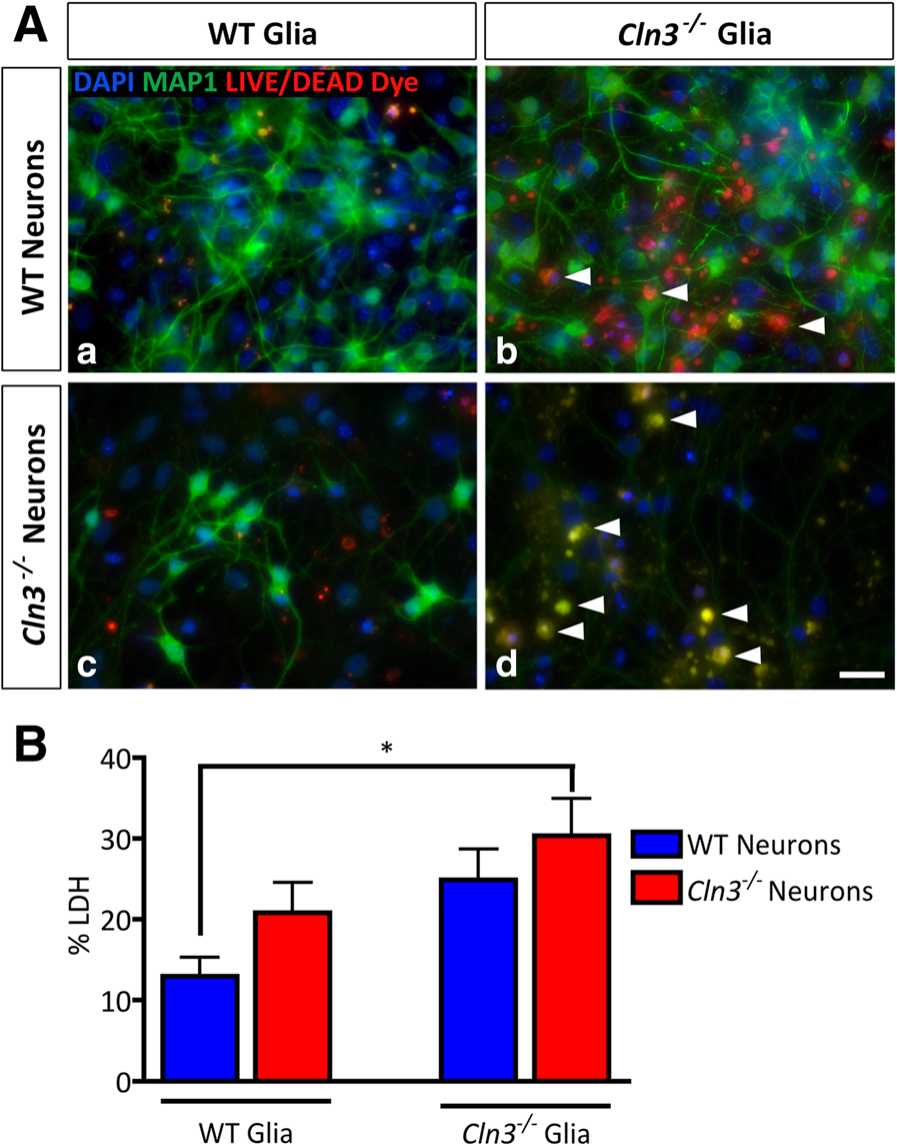
*Cln3* ^*−/−*^ cells negatively impact WT cells. P0 cortical wild type (WT) and *Cln3*-deficient (*Cln3* ^*−/−*^) neuronal cultures were combined with either WT or *Cln3* ^*−/−*^ mixed glia cultures to study the impact of these glial cells on neuronal health. **A** After 7 days of co-culture, WT co-cultures were healthy (**a**) but *Cln3* ^*−/−*^ mixed glia appeared to have a detrimental effect when cultured with both WT (**b**) and *Cln3* ^*−/−*^ neurons (**d**) with the latter being more dramatically affected. When WT mixed glia were co-cultured with *Cln3* ^*−/−*^ neurons, neuronal survival improved. **B** Significantly less LDH released was observed in WT neuron/WT mixed glia co-cultures compared to *Cln3* ^*−/−*^ neuron/*Cln3* ^*−/−*^ mixed glia co-cultures. Scale bar in (**A**) = 20 μm

Co-cultures were monitored for 7 days, and over this time, those composed of WT mixed glia and WT neurons remained healthy, with very few dying cells present (Fig. 12A, panel a). However, from day 2 onwards MAP2 immunoreactivity in processes of WT neurons grown with *Cln3* ^*−/−*^ glia became increasingly punctate, suggesting compromised neuronal health, and by day 7 many dying neurons with red nuclei were observed (Fig. 12A, panel b). The morphology of the surviving WT neurons was also dramatically altered, showing a reduction in soma size and neurite complexity (Additional file 6: Figure S5). The co-culture combination of *Cln3* ^*−/−*^ neurons with *Cln3* ^*−/−*^ glia was the most detrimental for neuronal health, since by day 7 most of the *Cln3* ^*−/−*^ neurons in these co-cultures were either dead (Fig. 12A, panel d), or appeared severely compromised, as judged by their morphology (Additional file 7: Figure S6). Interestingly, WT glia had a positive influence on both the survival and morphology of *Cln3* ^*−/−*^ neurons (Fig. 12A, panel c).

In these co-cultures, we observed that in the presence of *Cln3* ^*−/−*^ neurons*, Cln3* ^*−/−*^ astrocytes had smaller cell bodies and longer, more numerous processes (reminiscent of activated astrocytes in culture), when compared to *Cln3* ^*−/−*^astrocytes grown with WT neurons (Additional file 8: Figure S7). No such morphological change was evident when *Cln3* ^*−/−*^ neurons were co-cultured with WT glia, suggesting that *Cln3* ^*−/−*^ astrocytes are more sensitive to the environment than their WT counterparts. Under all culture conditions the morphology of microglia were heterogeneous with some cells bearing processes and others being fully rounded (data not shown).

These morphological findings correlated well with measurements of released LDH from the different co-cultures (Fig. 12B). The lowest LDH levels were observed when WT glia and neurons were co-cultured, but these levels increased dramatically when *Cln3* ^*−/−*^ glia were co-cultured with WT or *Cln3* ^*−/−*^ neurons (Fig. 12B). However, when *Cln3* ^*−/−*^neurons were co-cultured with WT, rather than *Cln3* ^*−/−*^ glia, a lower level of LDH release was observed, possibly due to the supportive influence of the WT cells (Fig. 12B). As might be expected, there was significantly more LDH released in *Cln3* ^*−/−*^ glia/*Cln3* ^*−/−*^ neuron co-cultures than in WT glia/WT neuron co-cultures (Fig. 12B).

These results suggest that *Cln3* ^*−/−*^ glia are detrimental to the health of both WT and *Cln3* ^*−/−*^ neurons, with *Cln3* ^*−/−*^ neurons being the most vulnerable. In contrast, WT glia appeared to have a positive influence on *Cln3* ^*−/−*^neurons, not just on survival, but also upon neurite complexity.

## Discussion

This study highlights the attenuated morphological transformation of astrocytes and microglia in both human and murine CLN3 disease. From studying cultured *Cln3* ^*−/−*^ astrocytes and microglia we have provided further support that their biology is impaired (see 16 [99]). Although specific microglial defects are certainly evident, astrocytes appear more severely affected, and these astrocyte defects may be due to the cytoskeletal abnormalities they display. Most importantly, we show that while *Cln3* ^*−/−*^ neurons are themselves compromised, the combined presence of *Cln3* ^*−/−*^ astrocytes and microglia exacerbate these phenotypes and have a detrimental effect on neuronal organization and health. Taken together, these data provide novel information that these glial cells exert a negative influence upon neurons and may directly influence neurodegeneration in CLN3 disease.

### Defects in glial biology could underlie components of CLN3 disease pathogenesis

Despite concerted efforts, the normal function of CLN3 remains poorly understood and it is unclear how its deficiency relates to cellular dysfunction, including that of astrocytes or microglia. Despite microglia accumulating large amounts of storage material, which is also present in astrocytes, the current view is that it is not the accumulation of storage material *per se* that directly causes cellular dysfunction and death. Instead it appears that other, as yet unknown, consequences of *Cln3*-deficiency are responsible. Our data suggest that these negative consequences of Cln3-deficiency are also evident in glia, rather than being confined to neurons, and it will be important to gain in vivo correlates of the data we have found in tissue culture.

Nevertheless, all the biological defects we found associated with cultured *Cln3* ^*−/−*^ astrocytes and microglia can plausibly be linked to known features of CLN3 disease pathogenesis, including the potential involvement of glutamate mediated excitotoxicity and oxidative stress. Indeed, although in vitro systems do not necessarily accurately reflect the in vivo situation, a series of similarities between our tissue culture observations and other reports exist. For example, the attenuated ability of *Cln3* ^*−/−*^ glia to respond morphologically to stimulation is also evident in the *Cln3* ^*−/−*^ mouse brain in vivo (Fig. 1, and [68, 69]), and a comparatively lower level of glial activation is evident in human CLN3 disease ([90], this study). This is in marked contrast to the robust glial activation and morphological transformation observed in all other forms of murine [24, 40, 47, 59, 64, 77, 93], ovine [61], or human NCL [90].

The impaired ability of *Cln3* ^*−/−*^ astrocytes to take up extracellular glutamate is consistent with the reduced expression of the glutamate receptor EAA2 in human CLN3 brain tissue [36], the reduced GLAST and glutamine synthetase levels evident in *Cln3* ^*Δex7/8*^ mice [16], and presynaptic elevation of glutamate in *Cln3* ^*−/−*^ mice [20]. Furthermore, neurons in these mice appear particularly vulnerable to AMPA-and NMDA-receptor stimulation [32, 43], perhaps because of excitotoxicity due to these elevated levels of glutamate, and different classes of glutamate antagonists provide some therapeutic benefit in *Cln3* ^*−/−*^ mice [41–44].

Another pivotal astrocyte function is the synthesis and secretion of the anti-oxidant glutathione (GSH) that plays a crucial role in protecting neurons against oxidative stress [31, 33]. Our data reveal that *Cln3* ^*−/−*^ astrocytes can still make, but fail to secrete, glutathione. Indeed, *Drosophila* lacking *CLN3* function are hypersensitive to oxidative stress [89], and oxidative damage has also been reported in both mouse [6] and human CLN3 disease [3].

Our calcium signaling studies revealed that *Cln3* ^*−/−*^ astrocytes fail to generate a calcium wave after exposure to ATP, providing further evidence that intercellular signaling between CLN3 astrocytes may be compromised [16], and it will be important to study calcium signaling in acute slice preparations. This could impact upon the control of neurotransmission in the JNCL brain and perhaps contribute to the seizure activity observed in this disease [58, 67, 88].

All these functional problems associated with *Cln3* ^*−/−*^ astrocytes and some of the phenotypes seen in vivo may at least partially be explained by their disrupted cytoskeleton, since expression of glutamate receptors at the cell surface [48], calcium signaling among astrocytes [27] and secretion by astrocytes [45] have all been shown to require a functional actin cytoskeleton. Indeed, the altered shape of *Cln3* ^*−/−*^ astrocytes, along with the difficulties they exhibit in changing their morphology in vivo and in our culture, may plausibly results from their disrupted cytoskeleton and it will be important to study this in more detail and determine their importance in vivo. How these defects in the cytoskeleton are related to *Cln3*-deficiency is unclear, but a functional interaction of CLN3 with non-muscle myosin-IIB has been reported [34], and a migration defect in *Cln3* ^*−/−*^ mouse embryonic fibroblasts that is consistent with our novel data for the impaired migration of *Cln3* ^*−/−*^ astrocytes.

### Alteration in protein secretion could impair cell-cell interactions

The protein secretion profiles of both *Cln3* ^*−/−*^ astrocytes and microglia was altered following activation, with astrocytes being more severely affected, showing significantly reduced levels of secretion of a range of proteins (Additional file 5: Tables S1 and S2). Our data are consistent with the reported evidence that LPS stimulation also results in a lower level of cytokine secretion by microglia derived from *Cln3* ^*Δex7/8*^ mice bearing the 1 kb deletion that is present in most CLN3 disease cases [99]. Intriguingly, these authors suggest that the responses of *Cln3*-deficient microglia are stimulus-dependent, with ceramide or neuronal cell lysates resulting in an increased inflammasome activation and expression of a wide array of proinflammatory cytokines and chemokines [99]. However, it should be borne in mind that these authors used *Cln3* ^*Δex7/8*^ ‘knock-in’ mice rather than the *Cln3* ^*−/−*^ mice used in our study, and this may influence the different phenotypes observed.

The biological significance of our data showing altered secretion profiles of *Cln3*-deficient glia remains unclear, but the downstream effects are likely to be complex given the multiple and possibly synergistic effects of secreted proteins on different cell types under both physiological and pathological situations [1, 19, 73, 74, 83]. It is also important to emphasize that what we have detected in vitro may not reflect the in vivo situation. Nevertheless, our data raise the possibility that cell-cell communication via secreted factors may potentially be perturbed in the CLN3 disease brain. In addition, many of the neuroprotective proteins routinely secreted by WT astrocytes after activation [15, 30, 57, 76, 95, 100], are also significantly reduced in cultures of activated *Cln3* ^*−/−*^ astrocytes, with two of these proteins (MCP-1 and RANTES) also being secreted at significantly lower levels by *Cln3* ^*−/−*^ microglia. The reduced expression of IL-6, RANTES and MCP-1, which can protect neurons against NMDA receptor-mediated excitotoxicity, may be especially relevant given the increased sensitivity of *Cln3*-deficient neurons to AMPA receptor-mediated excitoxicity [43, 65]. Thus, defects in glial-glial and glial-neuronal interactions have the potential to have a significant impact on neuronal health in CLN3 disease, a suggestion that prompted us to grow mixed glial co-cultures with neurons.

Until in vivo data regarding the relative levels of chemokines and cytokines become available, our in vitro data demonstrating altered secretion levels should be interpreted with caution, especially as these data come from pharmacologically stimulated cultures. However, the reduction in chemokine secretion by stimulated *Cln3* ^*−/−*^ glia in culture may also have a detrimental effect on the recruitment of microglia to sites of inflammation [72, 81], and partly explain the limited infiltration of monocytes and lymphocytes in CLN3 disease [50]. This reduced chemokine expression may also be associated with the attenuated microglial activation observed in vivo ([68, 69, 90], this study). Conversely, *Cln3* ^*−/−*^ astrocytes showed a reduced ability to secrete anti-inflammatory cytokines, such as IL-4, IL-10 and IL-2, which could also prove harmful. Both genetic and pharmaceutical approaches to attenuate the adaptive immune response have been shown to result in a significant improvement in the pathology of *Cln3* ^*−/−*^ mice [80].

### *Cln3*^*−/−*^ glia are detrimental to neuronal health

Defects in glial biology have been associated with neuronal dysfunction and loss in many neurodegenerative diseases, see [28, 29, 66, 75, 85]. Both positive and negative roles for astrocytes have been proposed, and recently, more active roles for astrocytes and microglia in directly influencing neuron survival have been postulated [49]. Using a co-culture approach, we have shown here that *Cln3* ^*−/−*^ astrocytes and microglia can indeed influence neuronal health, affecting the size and neurite complexity of both WT and *Cln3* ^*−/−*^ neurons, but also causing the death of the latter, which appear to be inherently compromised by *Cln3* deficiency. From our data it is not clear whether it is the *Cln3* ^*−/−*^ astrocytes or microglia, or a combination of both cell types that negatively influence neuronal heath. It has been suggested that astrocytes can be primed by microglia to become toxic to neurons [49], and it will be important to determine if similar mechanisms operate in CLN3 disease, especially in an in vivo context. However, it is apparent that despite any overt intrinsic survival defect in these short-term cultures, *Cln3* ^*−/−*^ neurons appeared to be compromised in terms of their morphology, and it will be important to investigate their functional status.

In other lysosomal storage disorders, introducing astrocyte-specific gene mutations is sufficient to harm neurons [21, 28], and correcting these defects is beneficial [102]. In our studies co-culturing *Cln3* ^*−/−*^ neurons with healthy glia improved many of their morphological defects, and resulted in an increase in their survival, suggesting that healthy glia appear to have a neuroprotective effect. This is in marked contrast to the apparently negative influence of *Cln3* ^*−/−*^glia upon both healthy and mutant neurons. It remains to be seen how accurately our data from cultures reflect the in vivo situation, and for this reason we have generated cell-type specific mutant mice in which we can inactivate *Cln3* in defined cell types in our future studies. Nevertheless, our data from this in vitro study suggests that therapies that target glia in addition to neurons may be an important step forward in treating this devastating disease.

## Conclusion

In summary, this study has provided evidence that both astrocytes and microglia derived from Cln3-deficient mice are dysfunctional, and this may contribute to directly harming neurons in this disorder. It will be important to investigate the underlying mechanisms and the extent of pathological involvement of each cell type in vivo. Given the close association between glial activation and neuron loss in these disorders, it will be important to determine whether glia also contribute to neuron loss in the other forms of NCL. This will information will be crucial for determining whether strategies that target glia will be of therapeutic value.

## Additional files

Additional file 1: Figure S1. Composition of Astrocyte Cultures. Primary cortical astrocyte cultures generated from P1–2 wild type (WT) and *Cln3*-deficient (*Cln3* ^*−/−*^) mice were grown for one week after the addition of Ara C, and in this example stimulated for a further 48 h before being immunostained with CD68 to identify microglia, O4 to identify oligodendrocytes, MAP2 together with NeuN to identify neurons and GFAP to identify astrocytes. DAPI was used to visualize all nuclei. WT and *Cln3* ^*−/−*^ astrocyte cultures contained few microglia or oligodendrocytes (A) and no neurons (B), and the vast majority of cells in *Cln3* ^*−/−*^ astrocyte cultures were GFAP-expressing astrocytes (C), with a higher proportion of DAPI + ve cells showing much weaker or no GFAP immunostaining in WT astrocyte cultures. Scale bar in (A) and (C) = 50 μm, and in (B) = 20 μm. (TIFF 13274 kb)

Additional file 2: Figure S2. Astrocyte Cultures stained with Glutamine Synthetase. Since GFAP expression can be down-regulated by astrocytes in culture, we also immunostained a parallel series of primary cortical astrocyte cultures from P1–2 wild type (WT) and *Cln3*-deficient (*Cln3* ^*−/−*^) mice with glutamine synthetase as an additional marker of astrocyte phenotype, after an additional 48 h in culture. DAPI was used to visualize all nuclei. Virtually all the DAPI stained cells (blue) were also immunoreactive for glutamine synthetase (red) in both WT and *Cln3* ^*−/−*^ cultures, and this was quantified as being 99.71 ± 0.15% (WT) and 99.29 ± 0.21% (*Cln3* ^*−/−*^) of the DAPI stained cells, respectively. Scale bar = 20 μm. (TIFF 2129 kb)

Additional file 3: Figure S3. Composition of Microglial Cultures. Primary cortical microglial cultures generated from P2–4 wild type (WT) and *Cln3*-deficient (*Cln3* ^*−/−*^) mice were immunostained with CD68 to identify microglia, O4 to identify oligodendrocytes, TuJ1 to identify neurons and GFAP to identify astrocytes. DAPI was used to visualize all nuclei. Practically all cells were CD68 expressing microglial cells (A), with virtually no cells expressing GFAP or O4 (B). Scale bar = 20 μm. (TIFF 8572 kb)

Additional file 4: Figure S4. LPS and INFγ induced signaling is not altered in *Cln3* ^*−/−*^ glia. Wild type (WT) and *Cln3*-deficient (*Cln3* ^*−/−*^) astrocytes were immunostained with GFAP and microglia with CD68. DAPI was used to visualize all nuclei. Few WT or *Cln3* ^*−/−*^ glia with nuclear-located P-p65 (A, C) and WT or *Cln3* ^*−/−*^ astrocytes with nuclear-located P-STAT1 (B) were observed under basal conditions, while the vast majority of both WT and *Cln3* ^*−/−*^ glia had P-STAT1 (B) and/or P-p65 (A, C) expressed in the nucleus upon stimulation. The percentage of cells expressing P-STAT1 and/or P-p65 in the nucleus was determined by counting 5 random fields per coverslip and a minimum of three coverslips per experiment. The means ±SEM shown are from three separate experiments. (TIFF 11278 kb)

Additional file 5: **Table S1.** Protein secretion profile of WT and *Cln3* ^*−/−*^ astrocytes under basal conditions. Differences between levels of secreted proteins in supernatants collected after 6 h, 24 h and 72 h from *Cln3* ^*−/−*^and WT astrocyte cultures grown under basal conditions. Data presented as % change (values from *Cln3* ^*−/−*^ astrocyte samples compared to corresponding WT astrocyte values) ± SEM from three biological replicates. (−) indicates proteins whose levels were below quantifiable detection levels. **Table S2**. Protein secretion profile of WT and *Cln3* ^*−/−*^ astrocytes after stimulation. Differences between levels of secreted proteins in supernatants collected from *Cln3* ^*−/−*^ and WT astrocytes after activation with LPS/IFNγ for 6 h, 24 h and 72 h. Data presented as % change (*Cln3* ^*−/−*^ astrocyte sample values compared to corresponding WT astrocyte values) ± SEM from three biological replicates. (−) indicates proteins whose levels were below quantifiable detection levels. (PDF 676 kb)

Additional file 6: Figure S5. An intact actin cytoskeleton is essential for glutathione secretion. To study the importance of the actin cytoskeleton for GSH secretion in astrocytes Cytochalasin D (1uM) was added to wild type (WT) astrocytes for 30 min prior to the start of the 8 h period over which the accumulation of secreted GSH in the medium was measured. Cells were then fixed and the actin cytoskeleton visualized with phalloidin. DAPI was used to visualize all nuclei. (A) Cytochalasin D clearly disrupted the F-actin filament organization in WT astrocytes. (B) Perturbing actin cytoskeletal polymerization significantly inhibited GSH secretion by WT astrocytes. Scale bar in (A) = 10 um. (TIFF 13687 kb)

Additional file 7: Figure S6. *Cln3* ^*−/−*^ mixed glia negatively impact neuronal morphology. Representative images of MAP2 expressing Wild type (WT) and *Cln3*-deficient (*Cln3* ^*−/−*^) neurons co-cultured with WT or *Cln3* ^*−/−*^ mixed glia are shown in (A) and quantification of neuronal soma size and neurite complexity under these different growth conditions are shown in (B-D). *Cln3* ^*−/−*^ neurons co-cultured with *Cln3* ^*−/−*^ mixed glia had a significantly smaller cell soma than did WT neurons co-cultured with WT glia (Aa, Ad, quantified in B). The substitution of WT mixed glia for *Cln3* ^*−/−*^ mixed glia significantly increased the soma size of *Cln3* ^*−/−*^ neurons (Ac, Ad, quantified in B). The total length of primary neurites was significantly reduced when *Cln3* ^*−/−*^ neurons were co-cultured with *Cln3* ^*−/−*^ mixed glia compared to when WT neurons were co-cultured with WT glia (Aa, Ad, quantified in C). The presence of *Cln3* ^*−/−*^ mixed glia also significantly reduced the length of the longest primary neurite in both WT and *Cln3* ^*−/−*^ neurons, and the length of the longest primary neurite was greater when WT neurons were co-cultured with WT glia than when *Cln3* ^*−/−*^ neurons were co-cultured with *Cln3* ^*−/−*^ mixed glia (C). The number of primary neurites (1. neurites that are extended from cell bodies) did not differ among the different co-cultures, but *Cln3* ^*−/−*^ mixed glia significantly reduced the number of both secondary neurites (2. neurites that branch off from primary neurites) and tertiary neurites (3. neurites that branch off from secondary neurites) in *Cln3* ^*−/−*^ neurons (D). The presence of *Cln3* ^*−/−*^ mixed glia also significantly reduced the number of tertiary neurites in WT neurons (D). Scale bar in A = 20 μm. (TIFF 5626 kb)

Additional file 8: Figure S7. Altered astrocyte morphology in co-cultures with *Cln3* ^*−/−*^ neurons. When co-cultured with *Cln3*-deficient (*Cln3* ^*−/−*^) neurons*, Cln3* ^*−/−*^ astrocytes (immunostained with GFAP, green) changed shape, having smaller cell bodies and longer more numerous processes (reminiscent of activated astrocytes in culture). No such change was observed when *Cln3* ^*−/−*^ astrocytes were grown with wild type (WT) neurons or when *Cln3* ^*−/−*^ neurons were grown with WT astrocytes. Scale bar = 20 μm. Nuclear stain DAPI (blue), Live/dead stain (red). (TIFF 10686 kb)
