# Supplementary figures and images for: Glial cells are functionally impaired in juvenile neuronal ceroid lipofuscinosis and detrimental to neurons
Source: Acta Neuropathol Commun. 2017 Oct 17;5:74. doi: 10.1186/s40478-017-0476-y (PMC5645909; doi:10.1186/s40478-017-0476-y)

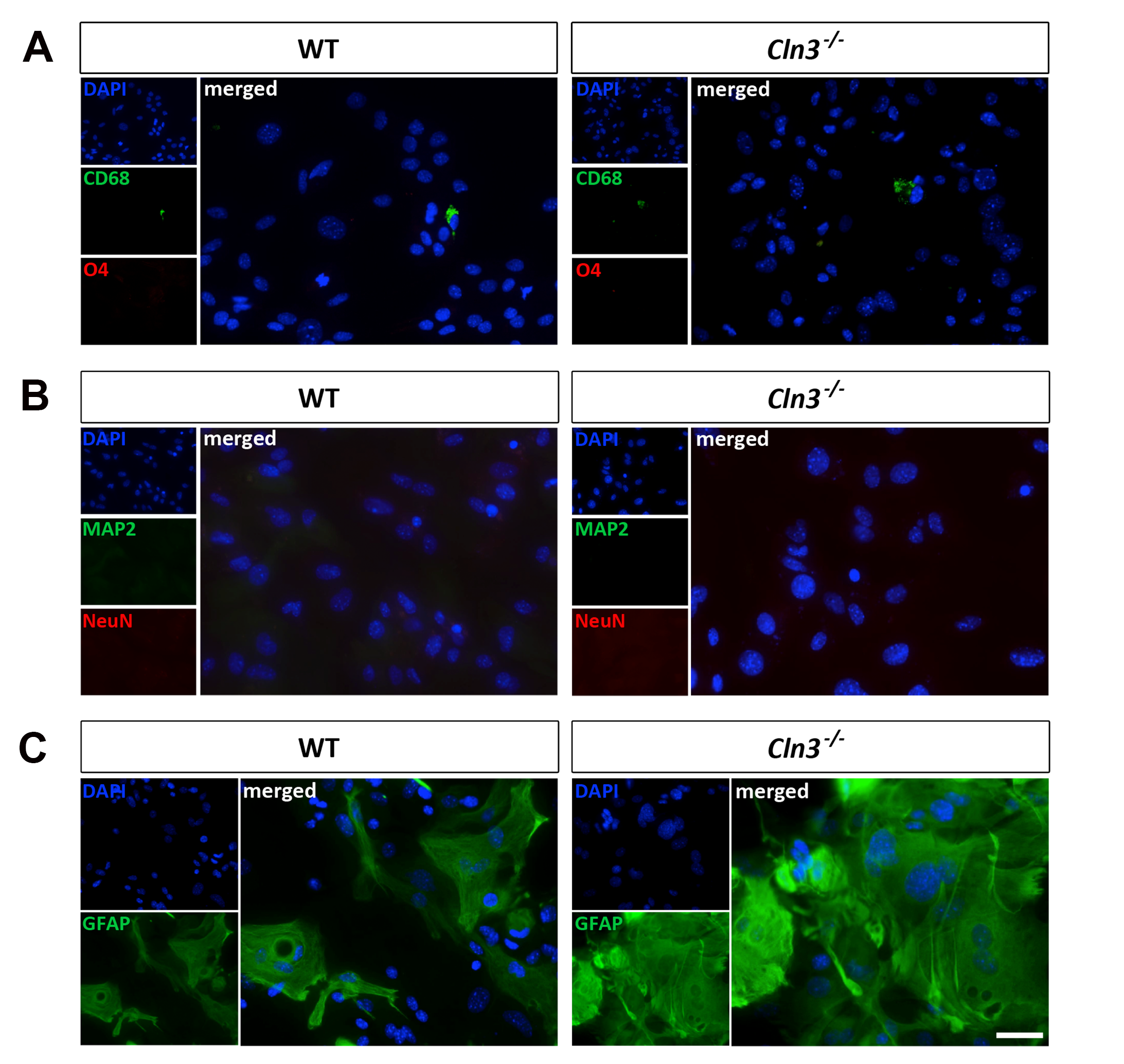

Supplement: Supplementary file 1 — Composition of Astrocyte Cultures. Primary cortical astrocyte cultures generated from P1–2 wild type (WT) and Cln3-deficient (Cln3 −/−) mice were grown for one week after the addition of Ara C, and in this example stimulated for a further 48 h before being immunostained with CD68 to identify microglia, O4 to identify oligodendrocytes, MAP2 together with NeuN to identify neurons and GFAP to identify astrocytes. DAPI was used to visualize all nuclei. WT and Cln3 −/− astrocyte cultures contained few microglia or oligodendrocytes (A) and no neurons (B), and the vast majority of cells in Cln3 −/− astrocyte cultures were GFAP-expressing astrocytes (C), with a higher proportion of DAPI + ve cells showing much weaker or no GFAP immunostaining in WT astrocyte cultures. Scale bar in (A) and (C) = 50 μm, and in (B) = 20 μm. (TIFF 13274 kb) [file 40478_2017_476_MOESM1_ESM.tif]

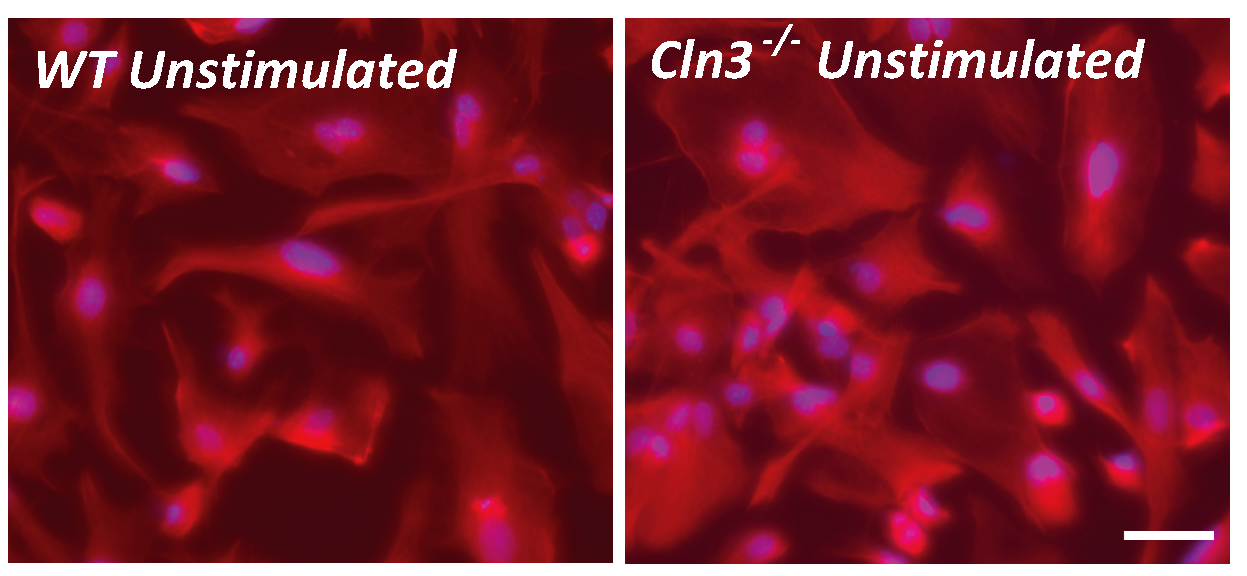

Supplement: Supplementary file 2 — Astrocyte Cultures stained with Glutamine Synthetase. Since GFAP expression can be down-regulated by astrocytes in culture, we also immunostained a parallel series of primary cortical astrocyte cultures from P1–2 wild type (WT) and Cln3-deficient (Cln3 −/−) mice with glutamine synthetase as an additional marker of astrocyte phenotype, after an additional 48 h in culture. DAPI was used to visualize all nuclei. Virtually all the DAPI stained cells (blue) were also immunoreactive for glutamine synthetase (red) in both WT and Cln3 −/− cultures, and this was quantified as being 99.71 ± 0.15% (WT) and 99.29 ± 0.21% (Cln3 −/−) of the DAPI stained cells, respectively. Scale bar = 20 μm. (TIFF 2129 kb) [file 40478_2017_476_MOESM2_ESM.tif]

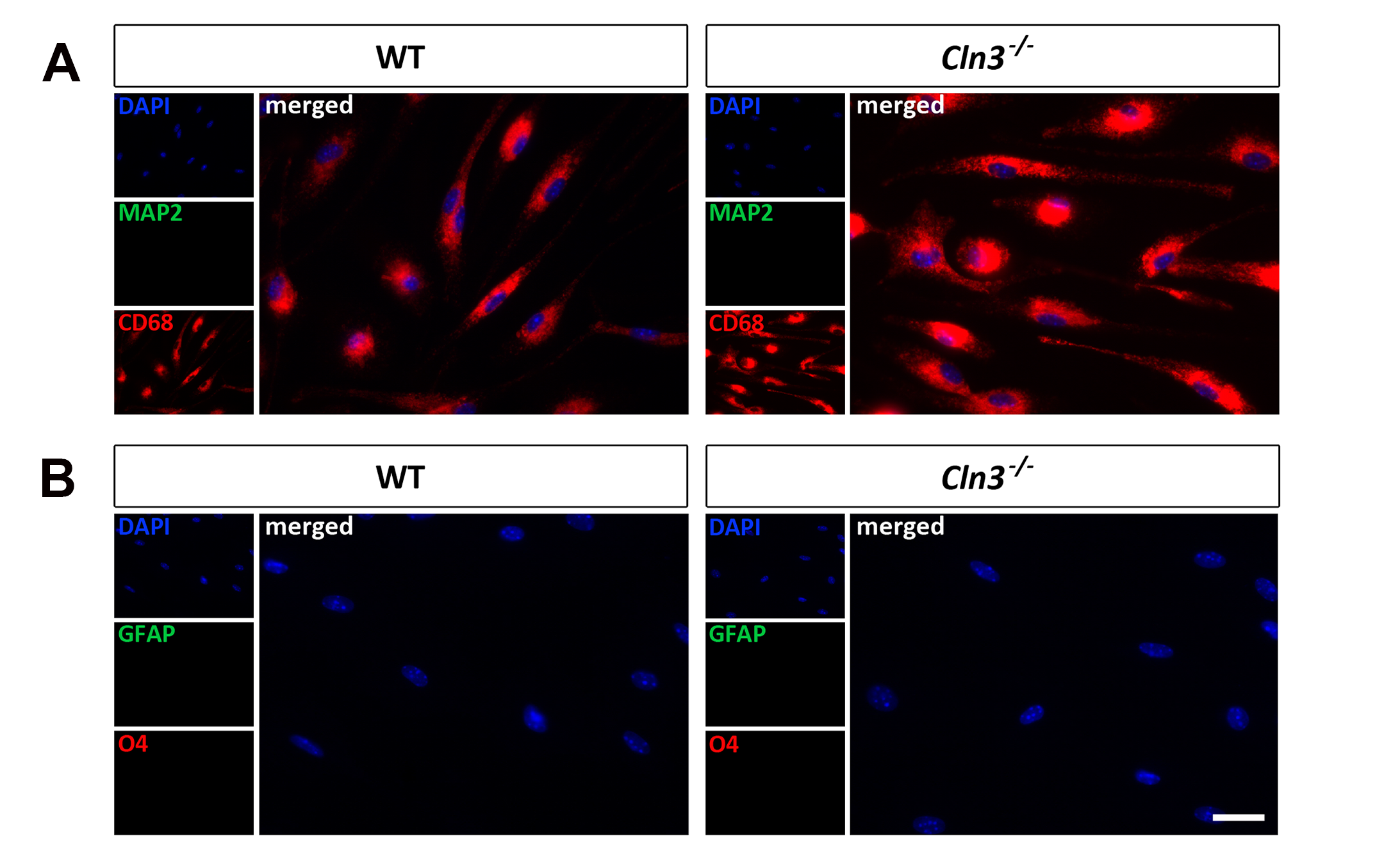

Supplement: Supplementary file 3 — Composition of Microglial Cultures. Primary cortical microglial cultures generated from P2–4 wild type (WT) and Cln3-deficient (Cln3 −/−) mice were immunostained with CD68 to identify microglia, O4 to identify oligodendrocytes, TuJ1 to identify neurons and GFAP to identify astrocytes. DAPI was used to visualize all nuclei. Practically all cells were CD68 expressing microglial cells (A), with virtually no cells expressing GFAP or O4 (B). Scale bar = 20 μm. (TIFF 8572 kb) [file 40478_2017_476_MOESM3_ESM.tif]

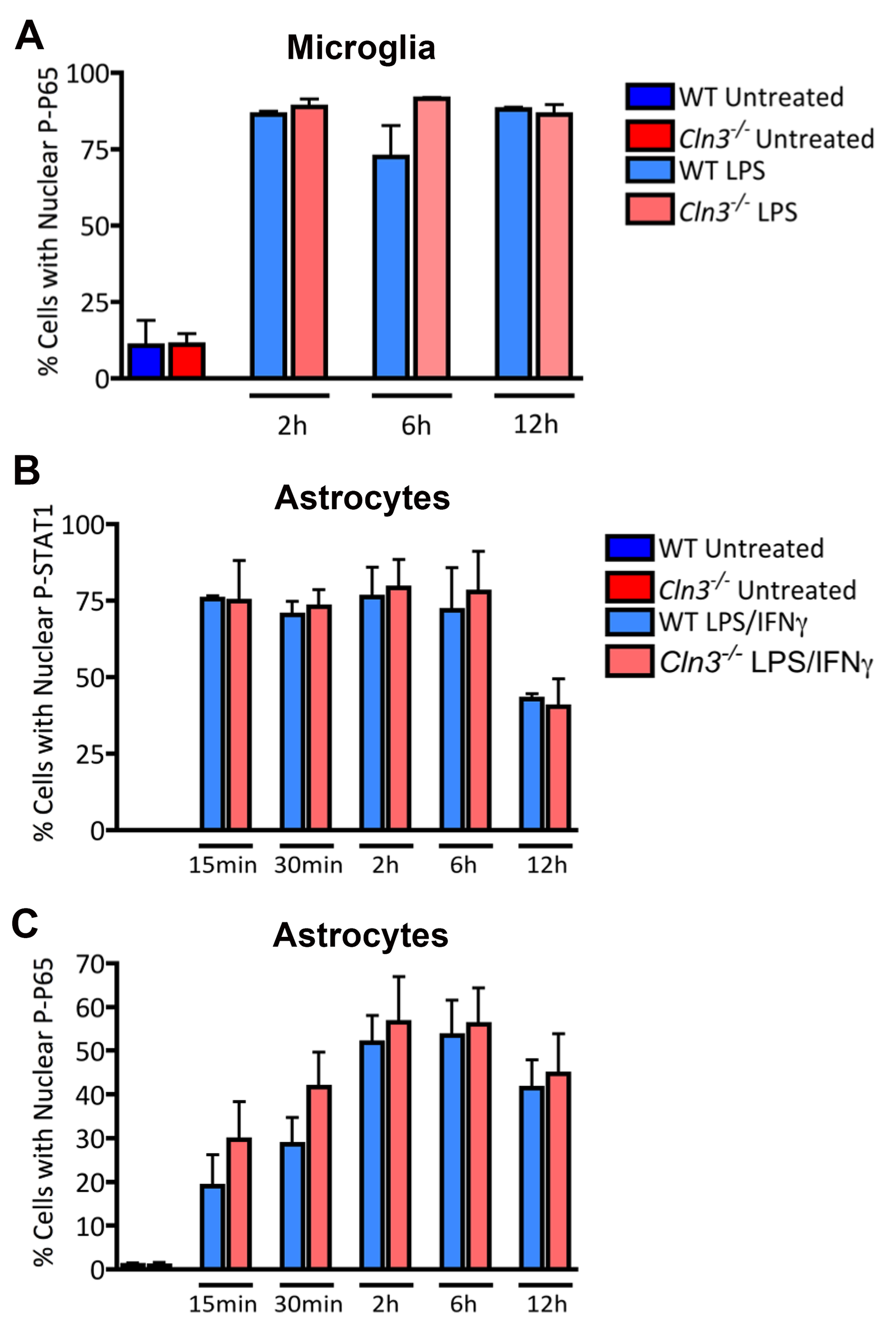

Supplement: Supplementary file 4 — LPS and INFγ induced signaling is not altered in Cln3 −/− glia. Wild type (WT) and Cln3-deficient (Cln3 −/−) astrocytes were immunostained with GFAP and microglia with CD68. DAPI was used to visualize all nuclei. Few WT or Cln3 −/− glia with nuclear-located P-p65 (A, C) and WT or Cln3 −/− astrocytes with nuclear-located P-STAT1 (B) were observed under basal conditions, while the vast majority of both WT and Cln3 −/− glia had P-STAT1 (B) and/or P-p65 (A, C) expressed in the nucleus upon stimulation. The percentage of cells expressing P-STAT1 and/or P-p65 in the nucleus was determined by counting 5 random fields per coverslip and a minimum of three coverslips per experiment. The means ±SEM shown are from three separate experiments. (TIFF 11278 kb) [file 40478_2017_476_MOESM4_ESM.tif]

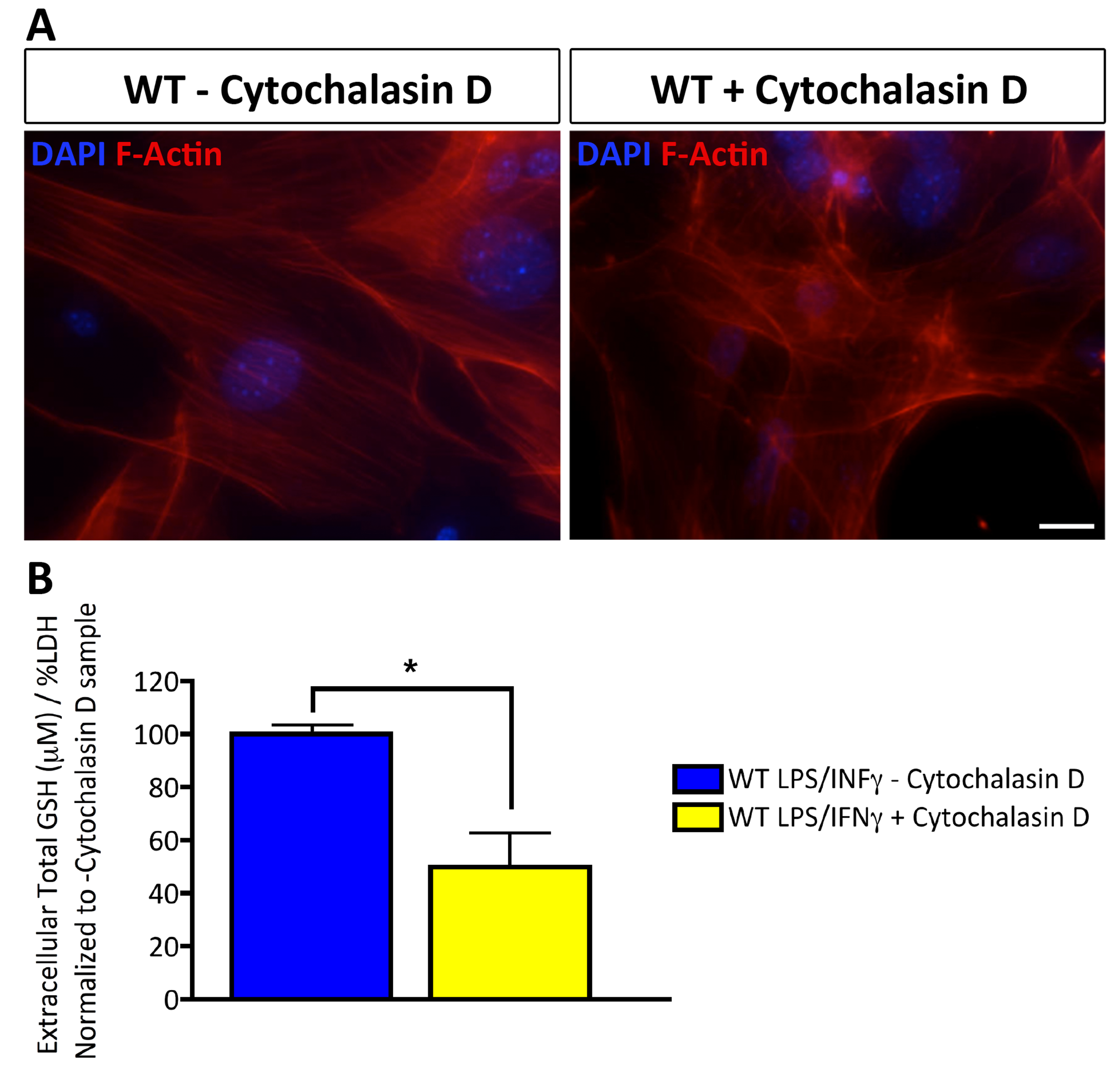

Supplement: Supplementary file 6 — An intact actin cytoskeleton is essential for glutathione secretion. To study the importance of the actin cytoskeleton for GSH secretion in astrocytes Cytochalasin D (1uM) was added to wild type (WT) astrocytes for 30 min prior to the start of the 8 h period over which the accumulation of secreted GSH in the medium was measured. Cells were then fixed and the actin cytoskeleton visualized with phalloidin. DAPI was used to visualize all nuclei. (A) Cytochalasin D clearly disrupted the F-actin filament organization in WT astrocytes. (B) Perturbing actin cytoskeletal polymerization significantly inhibited GSH secretion by WT astrocytes. Scale bar in (A) = 10 um. (TIFF 13687 kb) [file 40478_2017_476_MOESM6_ESM.tif]

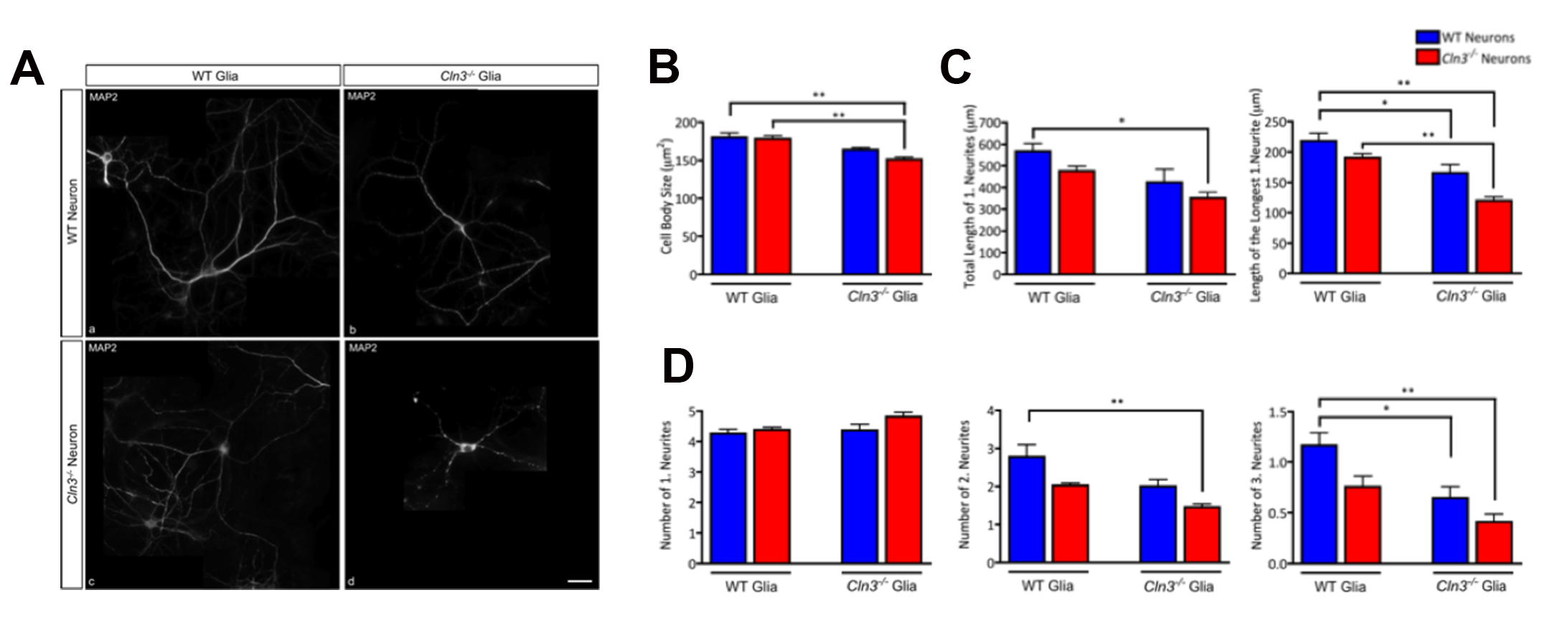

Supplement: Supplementary file 7 — Cln3 −/− mixed glia negatively impact neuronal morphology. Representative images of MAP2 expressing Wild type (WT) and Cln3-deficient (Cln3 −/−) neurons co-cultured with WT or Cln3 −/− mixed glia are shown in (A) and quantification of neuronal soma size and neurite complexity under these different growth conditions are shown in (B-D). Cln3 −/− neurons co-cultured with Cln3 −/− mixed glia had a significantly smaller cell soma than did WT neurons co-cultured with WT glia (Aa, Ad, quantified in B). The substitution of WT mixed glia for Cln3 −/− mixed glia significantly increased the soma size of Cln3 −/− neurons (Ac, Ad, quantified in B). The total length of primary neurites was significantly reduced when Cln3 −/− neurons were co-cultured with Cln3 −/− mixed glia compared to when WT neurons were co-cultured with WT glia (Aa, Ad, quantified in C). The presence of Cln3 −/− mixed glia also significantly reduced the length of the longest primary neurite in both WT and Cln3 −/− neurons, and the length of the longest primary neurite was greater when WT neurons were co-cultured with WT glia than when Cln3 −/− neurons were co-cultured with Cln3 −/− mixed glia (C). The number of primary neurites (1. neurites that are extended from cell bodies) did not differ among the different co-cultures, but Cln3 −/− mixed glia significantly reduced the number of both secondary neurites (2. neurites that branch off from primary neurites) and tertiary neurites (3. neurites that branch off from secondary neurites) in Cln3 −/− neurons (D). The presence of Cln3 −/− mixed glia also significantly reduced the number of tertiary neurites in WT neurons (D). Scale bar in A = 20 μm. (TIFF 5626 kb) [file 40478_2017_476_MOESM7_ESM.tif]

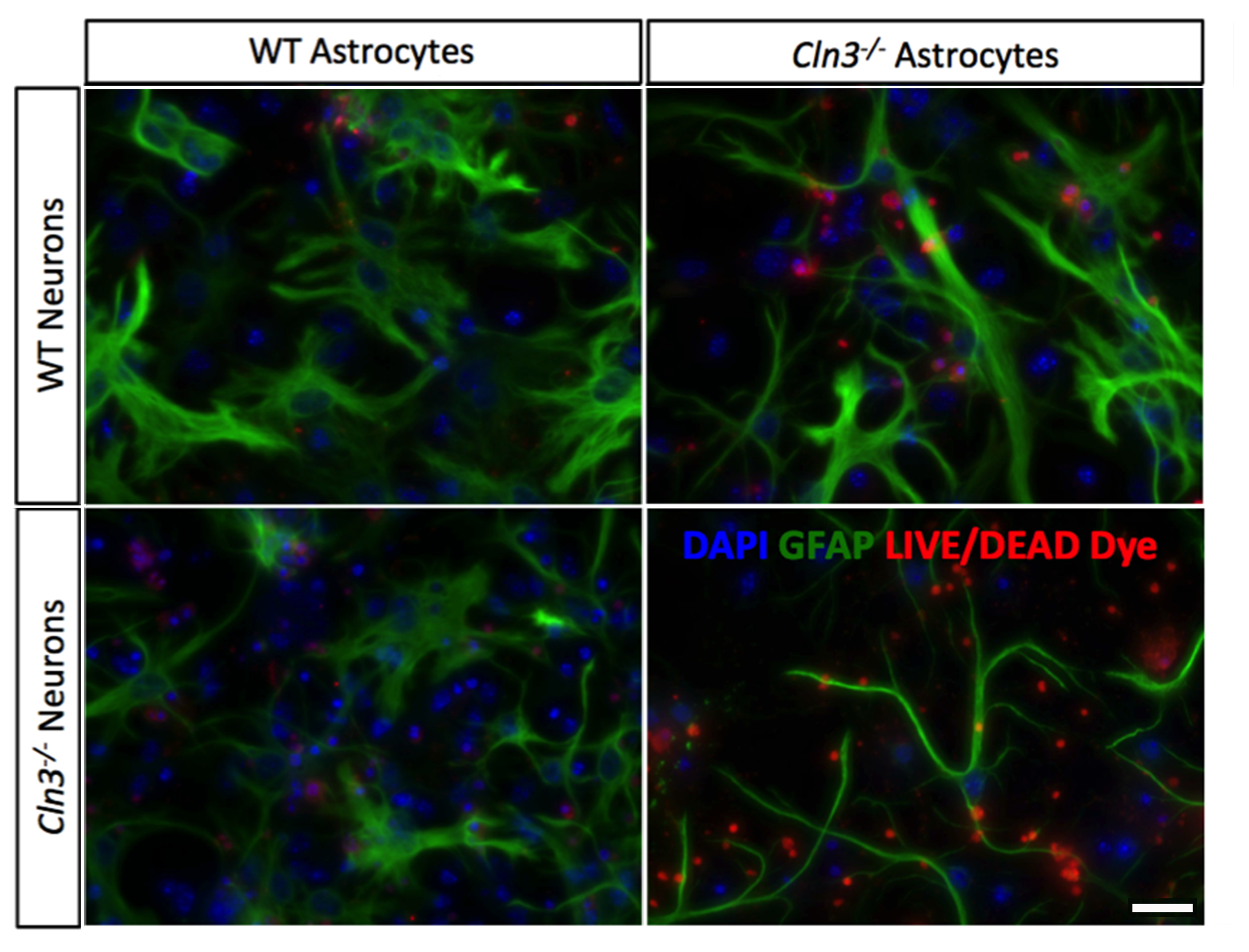

Supplement: Supplementary file 8 — Altered astrocyte morphology in co-cultures with Cln3 −/− neurons. When co-cultured with Cln3-deficient (Cln3 −/−) neurons, Cln3 −/− astrocytes (immunostained with GFAP, green) changed shape, having smaller cell bodies and longer more numerous processes (reminiscent of activated astrocytes in culture). No such change was observed when Cln3 −/− astrocytes were grown with wild type (WT) neurons or when Cln3 −/− neurons were grown with WT astrocytes. Scale bar = 20 μm. Nuclear stain DAPI (blue), Live/dead stain (red). (TIFF 10686 kb) [file 40478_2017_476_MOESM8_ESM.tif]
