# Supplementary material for: Glial cells are functionally impaired in juvenile neuronal ceroid lipofuscinosis and detrimental to neurons
Source: Acta Neuropathol Commun. 2017 Oct 17;5:74. doi: 10.1186/s40478-017-0476-y (PMC5645909; doi:10.1186/s40478-017-0476-y)
Supplement: Supplementary file 5 — Table S1. Protein secretion profile of WT and Cln3 −/− astrocytes under basal conditions. Differences between levels of secreted proteins in supernatants collected after 6 h, 24 h and 72 h from Cln3 −/−and WT astrocyte cultures grown under basal conditions. Data presented as % change (values from Cln3 −/− astrocyte samples compared to corresponding WT astrocyte values) ± SEM from three biological replicates. (−) indicates proteins whose levels were below quantifiable detection levels. Table S2. Protein secretion profile of WT and Cln3 −/− astrocytes after stimulation. Differences between levels of secreted proteins in supernatants collected from Cln3 −/− and WT astrocytes after activation with LPS/IFNγ for 6 h, 24 h and 72 h. Data presented as % change (Cln3 −/− astrocyte sample values compared to corresponding WT astrocyte values) ± SEM from three biological replicates. (−) indicates proteins whose levels were below quantifiable detection levels. (PDF 676 kb) [file 40478_2017_476_MOESM5_ESM.pdf]

Supplementary table 1

Mitogens	6h Untreated	24h Untreated	72h Untreated
CRP mouse	78.5%±27.3	14.2%±8.3	31.7%±4.1
M-CSF	18.5%±18.1	−32.2%±16.1	−41.5%±0.5 *
EGF	17.1%±6.7	26.5%±19.5	20.2%±11.8
IL-3	−5.3%±16.	14.0%±39.0	31.1%±29.1
FGF basic	−0.3%±24.9	0.4%±5.2	−25.5%±10.1
SCF	−20.6%±23.6	−64.3%±1.2	−57.3%±1.7
GM-CSF	−30.7%±14.2	−35.4%±2.1	27.9%±36.7
FGF-9	−21.4%±16.8	-	−31.5%±13.5
TPO	-	-	-
IL-5	-	-	-
**Pro-inflammatory**	**6h Untreated**	**24h Untreated**	**72h Untreated**
IFN γ	−13.9%±20.3	−40.7%±5.4	−22.8%±5.9
IL-17a	-	56.5.5%±0	−14.6%±13.4
Oncostatin M	−11.9%±17.1	−39.9%±12.2	−51.8%±0.9
IL-6	−57.4%±4.7	−72.9%±0.5	−68.6%±1.8
IL-12p70	-	−49.4%±7.9	-
IL-1 α	−29.6%±11.8	−48.6%±1.9	−45.3%±2.6
IL-18	152.9%±204.7	64.6%±69.0	-
TNF α	19.6%±16.8	−51.5%±5.0	−47.6%±6.3
CD40 ligand	-	-	-
IL-1 β	31.9%±33.9	−74.9%±25.1	0.7%±57.7
**Vascular/haematological**	**6h Untreated**	**24h Untreated**	**72h Untreated**
Tissue Factor	168.5%±76.4	237.6%±56.7	97.3%±84.5 *
Haptoglobin	3.2%±6.0	−15.3%±4.9	10.1%±14.8
VEGF	3.9%±26.7	1.2%±23.0	−0.3%±2.5
Endothelin	17.9%±25.0	4.8%±25.6	10.6%±72.2
Factor VII	4.9%±5.5	−8.2%±14.2	−8.9%±5.6
Fibrinogen	10.8%±7.4	7.6%±8.0	6.2%±1.0
MPO	-	-	-
SAP	-	-	-
vWF	-	-	-
**Chemokines**	**6h Untreated**	**24h Untreated**	**72h Untreated**
Eotaxin	39.2%±47.1	−44.7%±0	−55.9%±15.2
MIP-1 α	10.1%±2.8	−6.8%±11.8	−44.1%±3.6
MCP-3	−68.1%±5.3	−74.8%±6.7	−82.1%±0.5
MCP-1	−63.2%±5.8	−74.1%±7.4	−81.5%±0.5
KC/GRO α	-	-	−93.7%±0.2
Lymphotactin	8.9%±13.1	−40.2%±21.0	−41.9%±0.9
MIP-3 β	21.8%±8.5	−5.6%±11.2	−7.4%±5.7
MIP-2	−37.0%±6.1	−35.4%±2.1	−44.7%±3.2
IP-10	−58.8%±6.3	−77.3%±3.2	−62.7%±4.9
RANTES	−73.4%±5.3	−71.5%±3.4	−73.9%±1.7
MDC	45.2±39.4	−23.8±7.6	−45.5±7.5
MCP-5	−71.2%±2.6	−79.9%±1.5	−83.1%±1.5
MIP-1 γ	−78.3%±2.9	−84.5%±2.9	−83.0%±0.2 ***
GCP-2	-	−94.8%±1.0	−95.5%±16.7 ***
MIP-1 β	−45.9%±7.5	−66.4%±3.6	−80.7%±1.5
**Anti-inflammatory**	**6h Untreated**	**24h Untreated**	**72h Untreated**
IgG A	68.6%±50.8	16.6%±6.8	49.3%±16.0
IL-4	-	-	-
IL-10	-	-	-
IL-2	-	-	-
**Pleiotropic /others**	**6h Untreated**	**24h Untreated**	**72h Untreated**
LIF	−30.8%±4.7	−19.5%±11.7	−19.1%±3.9
TIMP1	10.1%±15.0	−62.5%±4.0	−61.8%±1.7 *
IL-11	−48.7%±3.8	−71.5%±0.1	−55.5%±1.6
IL-7	-	−23.9%±3.1	−34.4%±2.7
VCAM1	−54.4%±5.8	−47.3%±6.2	−46.0%±3.6
GST-α	-	-	-
CD40	-	-	-
MMP 9	-	-	-
Myoglobin	-	-	-
Apolipoprotein A-I	-	-	-
SGOT	-	-	-

Supplementary table 2

Mitogens	6h LPS/IFNY	24h LPS/IFNY	72h LPS/IFNY
CRP mouse	62.8%±44.5	82.3%±47.9 *	11.0%±15.6
M-CSF	60.9%±3.2	−14.3%±37.7	−21.1%±4.0 *
EGF	35.2%±2.8	29.1%±33.8	−31.6%±6.8 ***
IL-3	29.2%±10.8	−22.6%±37.8	−49.8%±3.1***
FGF basic	5.6%±13.0	−30.5%±17.6	−57.8%±2.1 ***
SCF	−3.4%±1.5	32.0%±49.0	−4.7%±3.5
GM-CSF	−3.4%±35.6	−20.3%±39.0	−88.1%±1.2 ***
FGF-9	−8%±11.8	−29.1%±30.6	−57.3%±1.1 ***
TPO	-	−61.4%11.8 **	−72.0%±4.7 ***
IL-5	-	-	-
**Pro-inflammatory**	**6h LPS/IFNY**	**24h LPS/IFNY**	**72h LPS/IFNY**
IFN γ	101.8%±67	34.2%±62.9	−92.4%±0.1 ***
IL-17a	18.8%±8.6	−59.5%±17.6 **	−80.3%±0.5 ***
Oncostatin M	16.2%±3.1	47.5%±53.6	4.0%±3.0
IL-6	−6.4%±12.4	−77.8%±10.9 ***	−93.9%±0.5 ***
IL-12p70	−9.7%±4.0	−44.3%±25.8	−72.3%±1.9 ***
IL-1 α	−14.9%±3.2	−6.0%±40.4	−70.0%±2.2 ***
IL-18	−40.6%±18.6	15.6%±74.5	−90.2%±4.9 **
TNF α	−43.8%±1.9	−53.3%±18.9 **	−71.4%±1.8 ***
CD40 ligand	-	28.8%±76.4	21.0%±50.0
IL-1 β	-	−66.3%±18.3	−53.8%±3.5 **
**Vascular/haematological**	**6h LPS/IFNY**	**24h LPS/IFNY**	**72h LPS/IFNY**
Tissue Factor	215.4%±79	−69.1%±12.2 **	−72.6%±6.2 ***
Haptoglobin	53.2%±23.1	143.0%±19.4	74.3%±26.7
VEGF	30%±6.4	−44.8%±25.1	−76.0%±1.9 ***
Endothelin	23%±19.1	−43.3%±28.4	−68.2%±4.4 ***
Factor VII	8.5%±6.8	1.8%±28.7	−42.4%±3.5 ***
Fibrinogen	5.0%±0.6	80.1%±31.7 ***	5.6%±5.0
MPO	-	-	-
SAP	-	-	-
vWF	-	-	-
**Chemokines**	**6h LPS/IFNY**	**24h LPS/IFNY**	**72h LPS/IFNY**
Eotaxin	73.5%±48.0	−71.03%±12.3 ***	−71.7%±2.8 ***
MIP-1 α	61.0%±3.2	−81.5%±8.0 **	−70.5%±1.1 ***
MCP-3	46.7%±54.5	−26.7%±35.1	−63.9%±2.3 ***
MCP-1	46.7%±4.8	−40.9%±28.5	−73.5%±1.4 ***
KC/GRO α	29.7%±32.8	−27.5%±35.6	−74.4%±3.7 ***
Lymphotactin	28.6%±6.0	40.4%±46.7	−3.6%±4.0
MIP-3 β	19.4%±5.7	−67.2%±10.5 ***	−73.2%±0.9 ***
MIP-2	19.3%±41.3	−51.2%±23.7 *	−86.4%±2.1 ***
IP-10	15.4%±8.4	−22.8%±29.4	−48.7%±5.6 ***
RANTES	−9.9%±22.6	−29.8%±33.7	−66.5%±3.4 ***
MDC	−22.5±12.6	−90.4±4.2 ***	−86.4±3.2 ***
MCP-5	−27.8%±12.6	−62.5%±17.8	−67.7%±0.9 ***
MIP-1 γ	−52.4%±6.5	−75.2%±11.6 ***	−55.0%±5.7 ***
GCP-2	−57.8%±16.7	−87.9%±5.8 ***	−84.4%±0.8 ***
MIP-1 β	−70.1%±2.4	−88.9%±4.8 ***	−81.7%±1.4 ***
**Anti-inflammatory**	**6h LPS/IFNY**	**24h LPS/IFNY**	**72h LPS/IFNY**
IgG A	123.8%±46.4	130.2%±70.5 **	49.7%±11.8
IL-4	57.9%±32.2	1.3%±31.1	−45.0%±3.5 ***
IL-10	36.1%±14.9	−48.3%±23.6 *	−75.4%±0.9 ***
IL-2	5.2%±6.6	−49.4%±23.8 **	−77.3%±0.7 ***
**Pleiotropic /others**	**6h LPS/IFNY**	**24h LPS/IFNY**	**72h LPS/IFNY**
LIF	40.1%±15.0	−31.8%±30.3	−72.3%±1.5 ***
TIMP1	32.1%±32.8	−57.9%±20.8 *	−62.5%±2.0 ***
IL-11	27.0%±6.9	−37.0%±26.7	−72.4%±4.8 ***
IL-7	6.5%±2.7	20.6%±41.9	−13.9%±1.4
VCAM1	−2.0%±9.1	−48.3%±22.3 **	−51.5%±2.8 ***
GST-α	-	−27.0%±30.1	−47.2%±15.7
CD40	-	−52.0%±21.4	−84.8%±2.9 ***
MMP 9	-	−85.1%±8.6 ***	−82.2%±0.3 ***
Myoglobin	-	-	-
Apolipoprotein A-I	-	-	-
SGOT	-	-	-
